# The Yin and Yang of Memory Consolidation: Hippocampal and Neocortical

**DOI:** 10.1371/journal.pbio.2000531

**Published:** 2017-01-13

**Authors:** Lisa Genzel, Janine I. Rossato, Justin Jacobse, Roddy M. Grieves, Patrick A. Spooner, Francesco P. Battaglia, Guillen Fernández, Richard G. M. Morris

**Affiliations:** 1 Centre for Cognitive and Neural Systems, University of Edinburgh, Edinburgh, United Kingdom; 2 Institute for Behavioural Neuroscience, University College London, London, United Kingdom; 3 Donders Institute for Brain, Cognition, and Behaviour, Radboud University and Radboud University Medical Centre, Nijmegen, The Netherlands; 4 Instituto for Neurosciencias, CSIC-UMH, San Juan de Alicante, Spain; Institute of Science and Technology Austria, Austria

## Abstract

While hippocampal and cortical mechanisms of memory consolidation have long been studied, their interaction is poorly understood. We sought to investigate potential interactions with respect to trace dominance, strengthening, and interference associated with postencoding novelty or sleep. A learning procedure was scheduled in a watermaze that placed the impact of novelty and sleep in opposition. Distinct behavioural manipulations—context preexposure or interference during memory retrieval—differentially affected trace dominance and trace survival, respectively. Analysis of immediate early gene expression revealed parallel up-regulation in the hippocampus and cortex, sustained in the hippocampus in association with novelty but in the cortex in association with sleep. These findings shed light on dynamically interacting mechanisms mediating the stabilization of hippocampal and neocortical memory traces. Hippocampal memory traces followed by novelty were more dominant by default but liable to interference, whereas sleep engaged a lasting stabilization of cortical traces and consequent trace dominance after preexposure.

## Author Summary

Memories are initially stored in a hippocampal–cortical network; however, which brain area is important for long-term storage depends on what happens after learning. For example, replay of recent memories during sleep is thought to lead to consolidation in the cortex. In contrast, postlearning novelty is thought to strengthen hippocampal memory traces via a mechanism that depends on dopamine.

Here, we show that indeed sleep leads to cortical consolidation, whereas novelty leads to hippocampal consolidation. Further, the memories followed by sleep or novelty differed in their behavioural expression and in the factors that could influence them. Memory traces followed by novelty were more dominant by default and showed stronger expression than those followed by sleep. This came at a cost: these memories were susceptible to interference that decreased their behavioural expression. In contrast, memories followed by sleep were more resistant to interference and benefitted from preexposure to the training context.

In sum, we showed that events that follow learning can influence the future expression of a memory trace.

## Introduction

Memory traces of episodic-like events are encoded in parallel by the hippocampus and neocortex throughout the day, but their retention over time is often transient. Traces subject to consolidation are retained, whereas later memory retrieval is unsuccessful when consolidation fails or is insufficient. Consolidation in both the hippocampus and neocortex is, however, now recognised as a complex set of processes involving both “cellular” mechanisms that operate largely within individual neurons and “systems” mechanisms that include network interactions across brain areas [1–4]. An additional mechanism called “reconsolidation” enables consolidated traces to be updated, indicating that stabilization need not imply fixation [5–7]. The distinction between cellular and systems consolidation is therefore not a sharp one, for the enactment of systems consolidation (involving interactions between hippocampus and neocortex) will necessarily involve the mechanisms of cellular consolidation as well. This overlap of mechanisms contributes to the challenge of studying of how hippocampal and cortical consolidation interact.

The overarching aim of this study was to investigate the interaction of hippocampal and cortical consolidation with respect to the retention of two potentially incompatible associations. Consider the following hypothetical situation. An experimental subject, be it human or an animal model, is required to learn first one thing and then later something different that may even contradict the first thing. In the procedural domain, it is important that the new skill overrides the first one and is then expressed in isolation (e.g., learning new balancing skills when riding a bicycle). However, in the episodic domain, it can be beneficial for the subject to remember both things even when they contradict one another (as in, “I used to think that John liked Mary but I now know it is only Mary that likes John”). This raises the conceptually deep issue of when new knowledge should interfere with and so “overwrite” earlier knowledge and when two items of ostensibly contradictory knowledge should both be retained.

Morris and Doyle [8] trained rats in a hippocampal-dependent watermaze task over many days to find a hidden escape platform in the northeast corner of the pool (in practice, this location was geometrically counterbalanced). Once this memory was well established, a “reversal” procedure was instituted such that, over eight trials, the platform was hidden in the opposite southwest corner. The key variable manipulated in the experiment was the interval of time between these eight trials (30 s or 24 h). In the 24-h condition, the animals learned the reversal and thereafter always searched for the platform in the southwest corner in successive memory tests over several weeks until the memory was lost. The animals also learned the reversal in the 30 s condition and first searched persistently in the southwest corner during an initial posttraining memory test, but, without any further training, they switched to searching preferentially in the northeast corner during a memory test conducted 12 d later. The amount of training on the reversal (eight trials) was exactly matched, but arguably the opportunity for engaging hippocampal (fast) and cortical (slow) consolidation mechanisms differed as a function of the short versus long intertrial intervals, respectively. In the former case, two incompatible traces were retained; in the latter, the first memory was overwritten.

The present study builds on this preliminary observation as a first step towards a systematic analysis of the intriguing issue of interference versus mutual retention. In the hippocampus, protein synthesis–dependent cellular consolidation acts soon after encoding as a selective filter to enable traces to be retained for longer [9–12], a process now known to be enhanced by postencoding novelty [13], possibly via a ventral tegmental area-hippocampal formation feedback loop [14]. Separately, cortical consolidation can occur (especially during overnight sleep) to guide and stabilize network interactions between the hippocampus and neocortex [15–22]. This likely operates using overlapping mechanisms (i.e., both cellular and systems mechanisms) but with the passage of sleep activating distinct neural mechanisms to enable either stable, episodic-like memory traces in the cortex, the potential loss of contextual associations, and/or the successful assimilation of new information with prior knowledge [23–27]. Cortical consolidation is widely thought to be a slower process [15], but there is growing evidence that it can sometimes be initiated soon after learning and act relatively quickly, such as during sleep [2,20,27,28].

We therefore sought to create two conflicting memory traces and then identify manipulations that would favour interference and loss or dominant behavioural expression of one contradictory memory without loss of the other. Postencoding novelty in the waking state or the opportunity for sleep soon after training were two distinct behavioural manipulations used to potentiate hippocampal or neocortical consolidation, respectively. These were supplemented by pretraining to assist assimilation with prior knowledge (cortical) or an interference protocol (likely to operate preferentially in the hippocampal domain).

One complication was that the novelty condition necessitated the simultaneous use of brief sleep deprivation to distinguish it from the sleep condition, and this necessitated an additional control study to check that brief sleep deprivation itself did not alter memory performance. Another unavoidable concern was that it is unlikely that novelty or sleep act exclusively on the hippocampus or cortex, respectively. Nonetheless, while less clear-cut than would be optimal, there are grounds for believing that postencoding novelty will have a preferential impact in the hippocampus [13,29], whereas sleep has a preferential impact on the interactions between the hippocampus and neocortex [16,17]. In the context of our experimental design, we can think of the two competing memory traces as occupying each side of a children’s “seesaw.” The relative dominance of one or the other trace is then “flipped” by changing behavioural parameters of training that likely affect hippocampal and cortical consolidation preferentially [30].

These manipulations being behavioural, it was incumbent upon us to identify whether potential neural markers of consolidation, such as immediate early gene (IEG) expression, were activated differentially at these two relevant anatomical sites. Our aim here was not to compare detailed patterns of expression across hippocampal subregions or cortical brain regions, nor to conduct a comprehensive comparison of expression patterns as a function of time [31], but rather to secure preliminary measures of the impact of these manipulations in the hippocampus and a specific region of the cortex. We predicted that novelty would lead to a learning-independent increase in immediate early gene (IEG) mRNA expression in the hippocampus related to the production of plasticity-related proteins implicated in synaptic tagging and capture [32] and the consequent consolidation of hippocampal traces [29]. In contrast, sleep should trigger a relatively selective increase in cortical consolidation after learning but against the background of a time-dependent down-regulation of IEG expression unrelated to memory consolidation, resembling findings recently reported for firing-rate changes [16,17,33–35].

## Results

**Fig 1:**
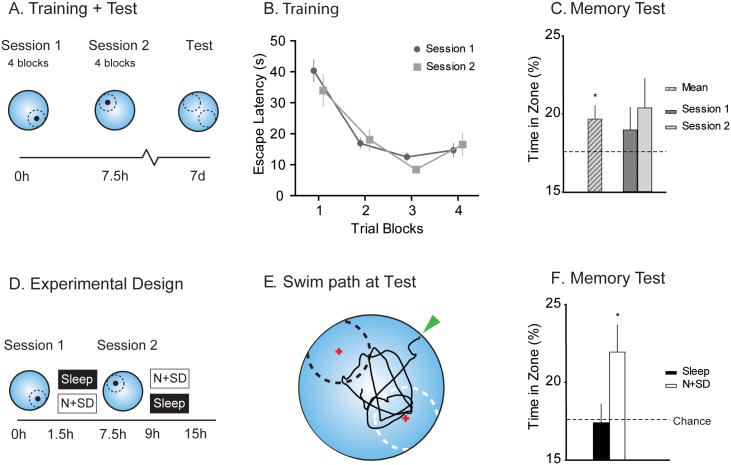
A watermaze protocol to examine competition of two memory traces. (A) Rats learned two opposite hidden platform locations in a watermaze over two successive sessions (four blocks of two trials per block, separated by 7.5 h) with a probe trial (no platform) conducted 7 d later. (B) Over both sessions, the animals decreased their escape latency (F = 21.5, df 1.7/51, *p* < 0.001). (C) At the 7 d memory test, the animals swam on average above chance level across zones (striped bar; t = 2.45, df 26, *p* = 0.022), but the trend favouring recency was not significant (*p* > 0.7). (D) Each session was followed by either sleep or novelty + sleep deprivation (N + SD) in a counterbalanced design. (E) Example swim path at test, with platform location followed by sleep NW (black zone) and N + SD SE (white zone) and starting location NE (green arrow). Note: based on extensive observation of swim patterns in the watermaze, the zones were deliberately designed to include an area by the side walls adjacent to one or other platform. (F) Swim time in zone followed by N + SD dominates over that followed by sleep (t = 1.97, df 52, *p* = 0.054, Cohen’s d = 0.54, N + SD to chance t = 2.31, df 26, *p* = 0.03). N + SD = novelty with sleep deprivation, NW = northwest, SE = southeast, *p < 0.05 *t*-test to chance. Means +/- 1 standard error of the mean (SEM). All raw data available in S1 Data.

In separate experiments, rats (*n* = 337) were randomly assigned for training in a spatial memory task, with brain tissue from a subset of animals analysed (blind) with respect to the expression of IEGs. An initial cohort (*n* = 32) was given brief spatial learning (four blocks of two trials per block) in a watermaze to one escape location, followed—after 7.5 h—by equivalent training to an opposite location (Fig 1A). These two sessions of training deliberately set up two competing memories such that memory tested much later could of be of one memory, the other, or of both. The animals learned each location in a comparable manner across the two training sessions within a day (Fig 1B) and showed, during a memory test 7 d later, significantly above chance swim time in predefined zones centred on the two platform locations (t = 2.45, df 26, *p* = 0.022; Fig 1C). While there was a trend favouring the more recently trained location, there was no significant difference in acquisition of memory associated with the two sessions (*p* > 0.7; Fig 1C).

Two conditions, (1) sleep and (2) novelty + sleep deprivation (N + SD), were scheduled in a counterbalanced and within-subjects manner after the first and second (competing) sessions of spatial learning. In the N + SD condition, the animals were placed in a novel environment with the repeated presentation of novel objects and other items and repeated gentle handling to prevent the animals from going to sleep. To control that the effects seen here were due to novelty and not sleep deprivation, we repeated the experiments but only with sleep deprivation by gentle handling and excluded novelty (see S11 Fig). We used a doubly counterbalanced design (with respect to both the order of platform location and of the N + SD versus sleep conditions; Fig 1D; S1 Fig). Accumulating data across animals and conditions required us to “rotate” the data matrix of half of the datasets by 180° for averaging, statistical, and graphical purposes. The swim paths in the 7 d probe test (memory retrieval) showed swim paths that moved back and forth between the two trained locations but revealed preferential search in the trained location that was followed by N + SD (representative animal in Fig 1E). The time searching in a virtual zone around the escape location was above chance for the N + SD condition but did not differ from chance in the sleep condition (N + SD t = 2.31, df 26, *p* = 0.03; sleep t = –0.13, df 26, *p* > 0.8, Fig 1F, separated for sequence [see S8 Fig]). Thus, under “baseline” conditions, and despite the opportunity for 6 further days of the animals’ routine sleep/waking cycle in the absence of further training, weak spatial learning followed by conditions favouring cellular consolidation in the hippocampus dominates the expression of memory in behaviour.

**Fig 2:**
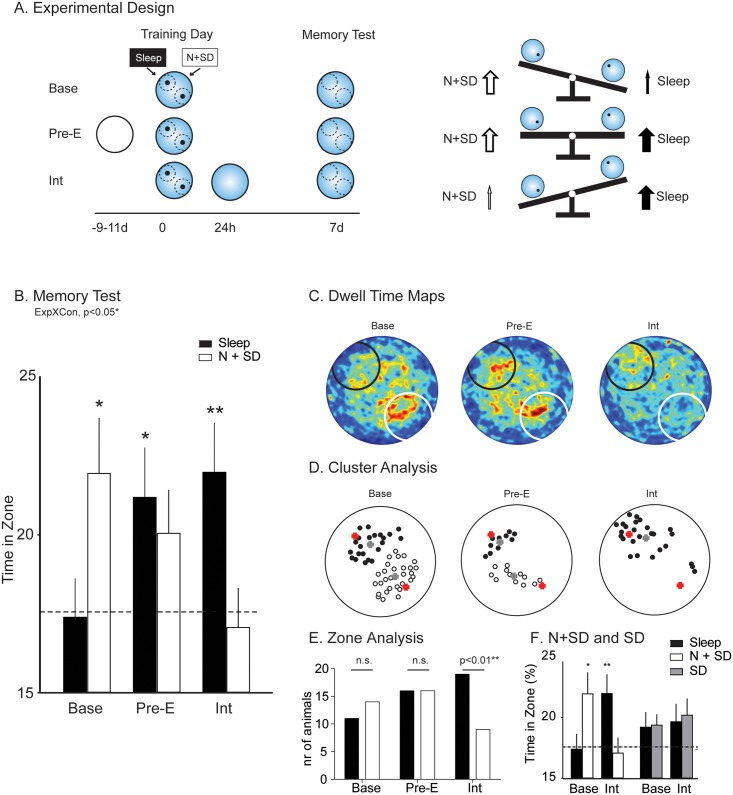
Distinct protocols favour cellular or systems consolidation. (A) The experimental design was, metaphorically, like a children’s seesaw. In addition to the baseline experiment (Fig 1), a further group was preexposed to the watermaze extramaze cues with a dry-land inlay for 3 d prior to the experiment (Pre-E) and a third group to an interference trial 24 h after training (Int). Sleep and N + SD followed in a counterbalanced manner. The “dominant” trace is the lightest, rising above the other conflicting trace. (B) In the zone analysis, a group x condition interaction was seen (F = 3.3, df 2/82, *p* = 0.043, with post hoc linear contrast *p* < 0.05, both controlling for sequence of consolidation-type, d = 3.37 *p = 0.03, *p* = 0.025 and **p = 0.01 *t*-test to chance). (C) Spatial dwell time maps of the watermaze at 7 d test with warm colours indicating higher average dwell time. Note systematic shift of the “hot” area across the different group protocols. Example paths from individual animals are shown in S18 Fig. (D) The cluster analysis showed two clusters in the baseline and preexposure groups but only one cluster in the interference group. Red cross indicates platform position, grey cross indicates cluster centre. Each point represents a local maxima derived from the dwell time maps. For panels C and D, the location followed by sleep is graphically presented at NW (black) and followed by N + SD at SE (white) but was counterbalanced. (E) Shows the number of animals that had above 20% swim time in the zone surrounding the platform location for each condition and experiment. Only in Int was a significant effect seen, with more sleep animals being above and more N + SD animals below chance level (Fisher’s exact test *p* = 0.019). (F) To ensure a specific effect of novelty, we ran two further cohorts (*n* = 32 in each) repeating the Base and Int experiments but this time with sleep deprivation by gentle handling instead of novelty (SD). There was a significant novelty x experiment x condition interaction (F = 3.7, df 1/116, *p* = 0.033). N + SD = novelty with sleep deprivation, NW = northwest, SE = southeast, Means +/- 1 SEM; all raw data available in S1 Data.

We then examined two further cohorts (*n* = 32 in each) in which we sought to flip the “seesaw” in one direction or the other. Experiment 1 can be thought of as having established a “default” situation in which the competing memory trace followed by N + SD is dominant over the memory trace followed by sleep (Fig 2A, Base condition). One behavioural method of flipping the seesaw was extensive preexposure of the animals to the context. Context preexposure would create prior knowledge of the extramaze cues of later watermaze learning and, we predicted, should enhance the speed and effectiveness of its cortical consolidation with relatively little effect on the trace, followed by N + SD (Fig 2A Pre-E). Context preexposure consisted of 3 d of 8 min exploration of the training context, achieved by placing the animals on a solid floor located within the watermaze (without water, but at the same height as the water would normally be, and with all extramaze cues visible). This allowed the animals to explore the environment and should have enabled them to learn about the relative location of extramaze cues. Subsequent training in the watermaze might then trigger learning in which the location of the hidden platform is rapidly assimilated within a previously established context representation. Specifically, we predicted “fast” systems consolidation [36], in much the same manner as can happen when animals have previously learned schematic knowledge [27].

The other method of influencing trace dominance was interference by removing access to the hidden platform during a test of memory 24 h after training. This involved a 120 s swim trial in the watermaze with no platform present, a procedure that would be likely to have the greatest effect on the “dominant” trace [30]. The consequences of such additional postacquisition learning should serve to diminish the capacity of the earlier trained N + SD trace to dominate the sleep memory with which it is competing (Fig 2A Int) and might even alter permanently the hippocampal representation of where escape may be possible.

The results confirmed these predictions (Fig 2B). Context preexposure enhanced performance after watermaze training to a level at which postencoding sleep enabled rapid assimilation of new information about the escape location during the four blocks of swim trials (Fig 2B). Conversely, postlearning interference had a greater impact on information that had been subject to strengthening by the prior N + SD condition (Fig 2B). Analysis of all three conditions—baseline, pre-exposure and interference—revealed a significant conditions x consolidation type interaction (F = 3.2, df 2/83, *p* = 0.043, d = 3.37 controlling for sequence of consolidation type).

This statistical interaction justified deeper analyses of the data—specifically, to compare the impact of masking (through trace dominance) and erasure (through interference or new learning). From both the baseline study and the additional experiments, we derived “dwell time” (heat) maps (Fig 2C) and a related but statistically distinct “cluster analysis” (Fig 2D). Dwell time maps show a summated occupancy of locations across the pool, with the “hot” colours reflecting greatest time. The cluster analysis, in contrast, identifies local maxima of occupancy even when these occur at levels well below the absolute maximum, signified by the hottest colour in the dwell time map. Using a gap-statistic method [37], the optimal number of clusters of occupancy could be calculated from the spread of all identified local maxima locations (see Methods). The cluster analysis is important as it has the potential to reveal the existence of a spatial memory (a focused cluster) even in circumstances in which its behavioural expression is masked by a separate dominant memory; similarly, it can reveal its absence. For graphical purposes, the sleep condition is displayed as northwest while the N + SD condition is shown as southeast (but this was fully counterbalanced, S1 Fig). For the baseline condition, the dwell time map (Fig 2C left) showed most searching in the N + SD location, complementing the previous zone-analysis, but the cluster-analysis (Fig 2D left) revealed memory for two separate locations. This indicated that the dominant behavioural expression of the memory consolidated by posttraining N + SD only masked the other memory with which it was in direct competition with respect to the control of behaviour. The presence of a significant negative correlation between swim time in the respective zones for N + SD and sleep offers further evidence for memory competition rather than erasure (S9 Fig). The preexposure condition revealed a more symmetrically balanced heat map and detectable clusters at or near both escape platform locations (Fig 2C and 2D, middle). In contrast, giving the animals 24 h interference trial, in which they could learn that neither platform was available, reduced the intensity of “hot” colours in the dwell time map and resulted in a complete loss of any clustering around the platform location followed by N + SD (Fig 2C and 2D, right). In this case, however, there are grounds for suspecting a loss of the N + SD location rather than masking because the cluster analysis identifies only one cluster centred on the sleep location with no local maxima for the N + SD being detectable. Our manipulations have had differential effects.

The distinction between “masking” and “erasure” is subtle but important. To further substantiate this putative dissociation, a third analysis was performed based on the swim time in the zones surrounding the platform locations (Fig 2E). For each experiment and group, we divided the animals into good performers above 20% swim time in zone and poor performers below it. The number of good and poor performers did not differ between the sleep and N + SD conditions for the Base and Pre-E experiment. In contrast, after interference, significantly more good performers were present in the sleep condition, and more poor performers were present in the N + SD condition (Fisher’s exact test *p* = 0.019). This supports the idea that the masking of memory traces takes place in the baseline experiment, but when the opportunity is given to learn that no escape is possible, memory erasure can both occur and occur selectively.

N + SD animals were placed in a novel environment and subjected to the repeated presentation of novel objects and items, coupled with gentle handling to prevent them going to sleep. To check whether the effects of N + SD were due to novelty, rather than sleep deprivation, two further cohorts (*n* = 32 in each) were run that repeated the Base and Int experiments but with sleep deprivation by gentle handling and explicitly excluding novelty (see Fig 2F and S11 Fig). In contrast to the N + SD experiments, sleep deprivation by gentle handling did not lead to the interaction effects seen across experiments and conditions. In fact, when compared with the main experimental condition, there was a significant novelty (N + SD/SD) x experiment (Base/Int) x condition (sleep/sleep deprivation) interaction (F = 3.7, df 1/116, *p* = 0.033).

The next important step was to complement these behavioural observations with measures of likely markers of consolidation, namely immediate early genes. If the account we have offered so far in terms of differential impact of hippocampal versus cortical consolidation is valid, there should be analogous changes at the IEG level following these same manipulations. We therefore sought to observe the impact of N + SD and sleep on genes likely relevant to neuronal plasticity. We focused on selected markers to represent activity-related and plasticity-related processes: immediate early gene expression of *cFos* mRNA as an indicator of activity [38] and *Zif-268 and Arc* mRNA as an indicator of plasticity [1]. These were all monitored in both the hippocampus and medial prefrontal cortex (mPFC).

**Fig 3:**
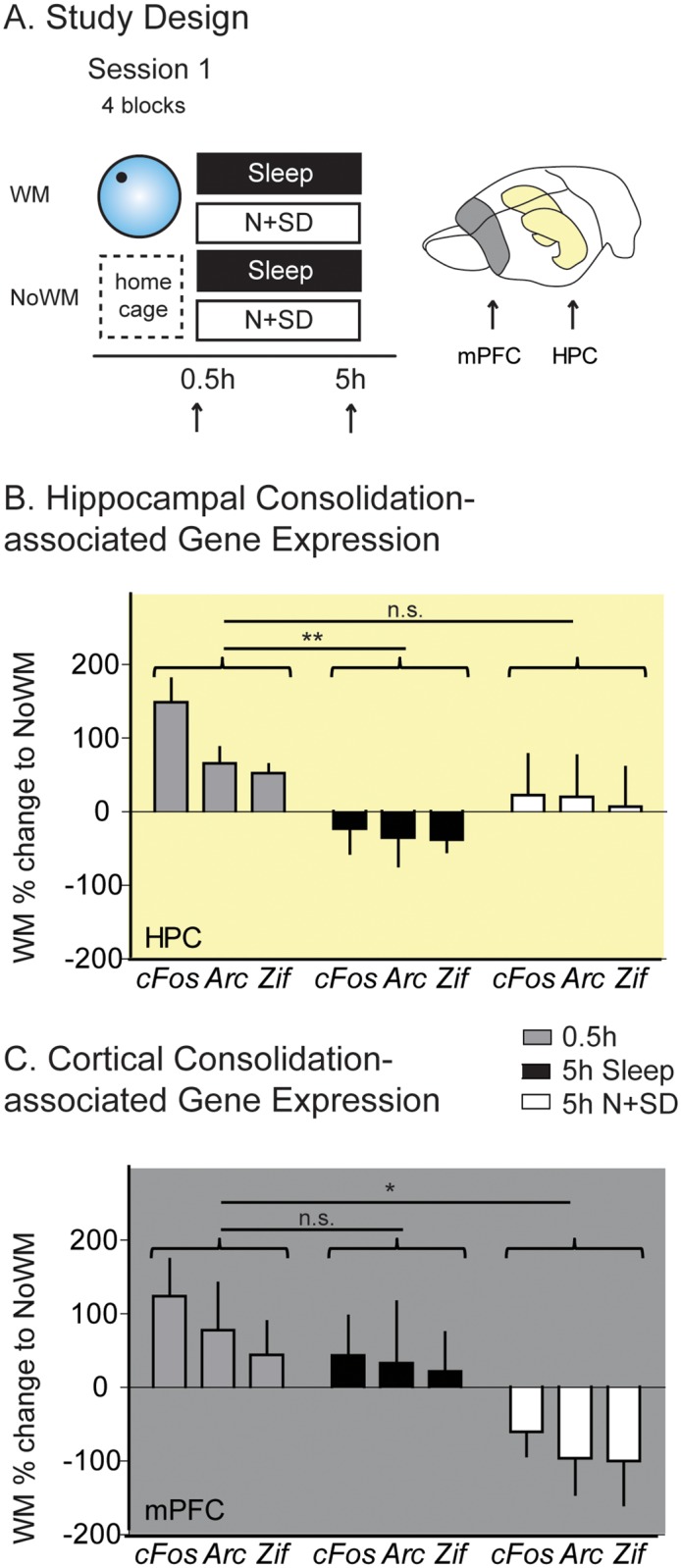
RT-qPCR analysis consolidation. (A) To elucidate learning specific effects, we directly compared animals 0.5 h and 5 h after having a learning session in the watermaze (WM) or remaining in the home cage (no exposure to the watermaze [NoWM]) with either sleep or N + SD in the 5 h period. Positive values reflect higher gene expression in WM animals, and negative values reflect higher gene expression in NoWM. At encoding (0.5 h), a significant gene effect was seen, with *cFos* showing higher gene expression changes than both other genes across both brain areas (F = 19.8, df 1.1/6.6, *p* = 0.01, post hoc simple, contrasts *cFos* versus *Arc* F = 10.3, df 1/4, *p* = 0.033 and *cFos* versus *Zif* F = 56.5, df 1/4, *p* = 0.002). 5 h later (Sleep and N + SD), a significant brain area x condition interaction was seen (F = 6.9, df 1/24, *p* = 0.015, post hoc linear contrast *p* = 0.015), with WM showing higher gene expression than NoWM in the HPC after N + SD, but lower in sleep. Thus, time (0.5 and 5 h) showed a differential effect across brain areas and condition (B and C). (B) In the hippocampus, only sleep and not N + SD showed a significant decrease in gene expression from 0.5 to 5 h (Sleep: F = 12.2, df 1/8, *p* = 0.008, d = 2.48; N + SD: F = 1.6, df 1/8, *p* > 0.2, d = 0.90). (C) In contrast, in the mPFC, the opposite pattern was seen, with a significant decrease in gene expression only in N + SD (sleep: F = 0.3, df 1/8, *p* > 0.5, d = 0.42; N + SD: F = 5.6, df 1/8, *p* = 0.046, d = 1.67). HPC = hippocampus, mPFC = medial prefrontal cortex, IEG = immediate early gene, N + SD = novelty with sleep deprivation. Means +/- 1 SEM. All raw data available in S1 Data.

**Fig 4:**
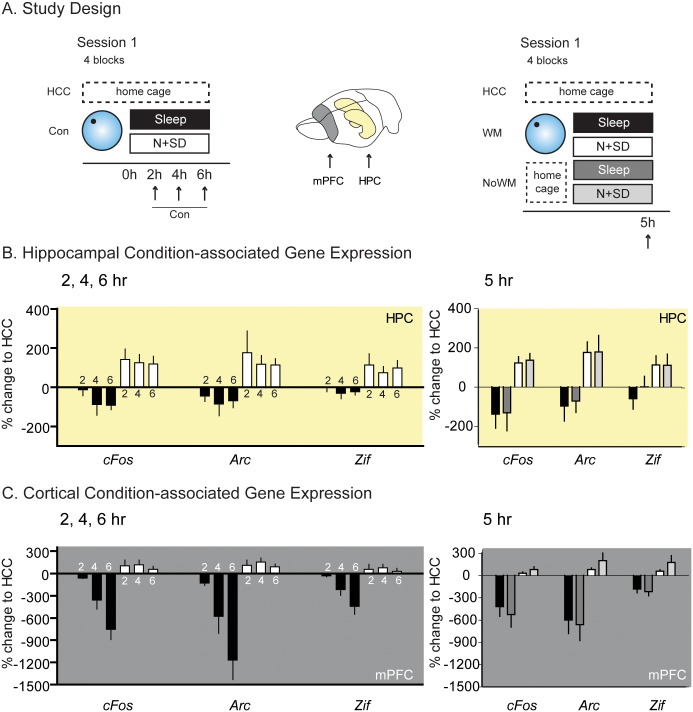
RT-qPCR analysis conditions. (A) Left: Rats learned one platform location in the watermaze followed by either sleep or N + SD. The animals were humanely killed (arrows) at different time points throughout the procedure, capturing consolidation steps across the two conditions (Con) to allow for qPCR analysis of IEG expression in the mPFC and HPC. Right: To exclude learning-specific effects, we directly compared animals at 5 h after having a learning session in the watermaze (WM) or remaining in the home cage (NoWM) with either sleep or N + SD in the 5 h period. All animals are compared to home cage controls (HCC). (B) In comparison to a neutral wake condition (HCC), N + SD showed elevated gene expression that was sustained throughout the N + SD period in HPC (yellow background). In contrast, sleep showed a decrease. There was both a condition x brain area and condition x time interaction seen during the consolidation period (F = 13.1, df 1/24, *p* = 0.001 with post hoc linear contrast *p* < 0.001; F = 6.1, df 2/24, *p* = 0.007, respectively). These effects were seen independent from any experiences in the watermaze (right). (C) In the mPFC, sleep was associated with a decrease in IEG, which was monotonic with respect to time in the mPFC (grey background; 2, 4, and 6 h from left to right). In contrast, N + SD showed an up-regulation of expression. And again, these effects were seen independent from any experiences in the watermaze (right). HPC = hippocampus, mPFC = medial prefrontal cortex, IEG = immediate early gene, N + SD = novelty with sleep deprivation, HCC = home cage controls. Means +/- 1 SEM. All raw data available in S1 Data.

There were two key decisions about the experimental design. First, the experimental method involved in measuring mRNA expression in association with memory encoding and consolidation was real-time quantitative PCR analysis. We chose qPCR in contrast to immunocytochemistry as we sought to achieve an exact and quantitative measure of the extent and time course of gene transcription. This enabled more complex statistical models with multiple within- and between-subject comparisons at the cost of being unable to compare and contrast different subregions within the hippocampus or areas of the cortex. Second, behavioural training was necessarily to a single escape location in the watermaze because the “trace-competition” design could not be used unambiguously. Additionally, it was important to measure IEG activation at defined time points soon after encoding, and this also precluded the use of training to two escape platforms several hours apart. The consolidation-specific effects of the two conditions (sleep and N + SD) were investigated as well as general effects of the condition. To achieve this aim, specific contrasts in our analysis were chosen. For consolidation-specific effects, the contrast was between animals that did or did not experience the watermaze, but we controlled for their condition with both groups having either sleep or N + SD (Fig 3). In contrast, for general condition effects, the contrast was between animals that experienced the watermaze (and had either sleep or N + SD) with awake, home cage control animals (Fig 4).

The study design focused on comparing IEG expression during the course of the respective postencoding N + SD or sleep manipulations in brain tissue from animals that had all learned the watermaze in a single session with tissue from animals that had not been subject to training (Fig 3). For all experiments, the brains were extracted with the hippocampus (HPC) (yellow) and mPFC (grey) immediately dissected and then snap-frozen for later analysis (Fig 3A). We compared brain tissue from animals that had experienced a learning session in the watermaze (WM) with animals that did not (NoWM; Fig 3B and 3C). This was done either directly after encoding (0.5 h) or 5 h into the consolidation period in association with postencoding sleep or N + SD (*n* = 30). The graphs are plotted such that positive values indicate higher gene expression in WM than in NoWM (negative values, vice versa). We chose a neutral wake control condition (NoWM) because possible alternative control conditions such as swimming in the watermaze without a platform can display alterations in IEG expression in association with stress or with incidental learning about the environment through exploration [39–41], and these confounding factors can hinder interpretation of results [42]. Furthermore, for present purposes, the critical results are the contrasts between N + SD and sleep with respect to spatial learning or its absence.

For the 0.5 h encoding condition, without subsequent behavioural manipulations, there was a significant and equivalent increase gene expression in both the HPC and mPFC relative to NoWM (Fig 3B and 3C, left). There was a significantly larger increase in *cFos* compared to the plasticity-associated genes (F = 19.8, df 1.1/6.6, *p* = 0.01, post hoc simple, contrasts *cFos* versus *Arc* F = 10.3, df 1/4, *p* = 0.033 and *cFos* versus *Zif* F = 56.5, df 1/4, *p* = 0.002). This is an important finding, as it points to substantial and rapid gene up-regulation in both brain regions at or soon after learning.

The comparison of WM and NoWM after 5 h of N + SD or sleep revealed different patterns of gene expression in hippocampus versus cortex (Fig 3B and 3C, right). In the hippocampus, the pattern was for higher gene expression with WM after N + SD and lower after sleep; in the cortex, the opposite pattern prevailed. The ANOVA showed a significant brain area x condition interaction at the 5 h time point (F = 6.9, df 1/24, *p* = 0.015, post hoc linear contrast *p* = 0.015). This interaction is important and most easily illustrated by focusing on the effect of time (0.5 and 5 h) on *cFos*. Note that the relative level of gene expression in HPC after 5 h of N + SD (at approximately 20% greater for the animals had been trained) was not significantly less than that observed at 0.5 h (t = 2.0, df = 8, *p* > 0.08); a much larger and now significant decrease in relative gene expression over time was observed for sleep with gene expression being approximately 35% less in animals that had been trained (t = 4.0, df = 8, *p* = 0.004; Fig 3B). In contrast, if one looks at the relative impact of N + SD and sleep in cortex, the decrease in relative *cFos* expression across training conditions from +120% to –60% for N + SD was significant (t = 3.1, df = 8, *p* = 0.015), whereas for sleep the corresponding difference did not reach significance (t = 1.1, df = 8, *p* > 0.3; Fig 3C). The same pattern was also seen across all genes (HPC: sleep: F = 12.2, df 1/8, *p* = 0.008, d = 2.48; N + SD: F = 1.6, df 1/8, *p* > 0.2, d = 0.90; mPFC: sleep: F = 0.3, df 1/8, *p* > 0.5, d = 0.42; N + SD: F = 5.6, df 1/8, *p* = 0.046, d = 1.67).

The differential effects of N + SD and sleep on gene expression, offset by consolidation-associated changes, warranted further study into the more general effects of N + SD and sleep. Specifically, in a separate cohort of 35 animals, IEG expression was measured in hippocampus (yellow) or prefrontal cortex (grey) at 2 h, 4 h, or 6 h after encoding (Fig 4A), during which postencoding N + SD (white) or sleep (black) were occurring in different subgroups of animals (conditions). This time, these were referenced to a neutral, awake home cage control (HCC) to enable comparison of the general change in gene expression across both behaviours (*n* = 5 for each “con” subgroup and *n* = 5 for the “HCC” control). In the separate cohort of trained and untrained animals (*n* = 25), the time-point 5 h was examined only.

Animals allowed to sleep for varying time periods showed a monotonic, time-dependent decrease in gene expression relative to HCCs at the neocortical but not the hippocampal site (for an explanation of the “fold-change” measure, see Methods RT-qPCR). In contrast, the impact of recurring exposure to novelty coupled to sleep deprivation was associated with positive changes in gene expression in both brain regions that did not change over time. An overall ANOVA of gene expression—including condition (Sleep, N + SD), time (2, 4, and 6 h), brain area (HPC, mPFC), and gene (*cFos*, *Zif-268* and *Arc*)—revealed significant interactions for condition x time and condition x brain area (F = 13.1, df 1/24, *p* = 0.001 with post hoc linear contrast *p* < 0.001; F = 6.1, df 2/24, *p* = 0.007, respectively). The same general pattern was seen for all genes, but *Arc* expression drove the result in the cortex as indicated by a significant condition x time x brain area x gene interaction. Ongoing novelty for 2, 4, or 6 h was associated with an apparently stable up-regulation of IEG expression with no relevant interactions.

In an additional control analysis, we also compared the 5 h WM/NoWM and sleep/N + SD conditions to a neutral wake home cage control and could replicate the general decrease in gene expression in sleep and increase in N + SD, which was thus shown to be learning independent and mainly reflected the current experience (sleep or novelty) of the animal (Fig 4 right).

## Discussion

The overarching aim of this study was to investigate the interaction of hippocampal and cortical consolidation with respect to the retention of two potentially incompatible associations. The key findings are that (1) rats can learn and retain two incompatible spatial memories without interference, with one memory dominating expression in certain circumstances; (2) postencoding sleep or novelty plus sleep deprivation have differential effects on memory consolidation and the resulting pattern of behavioural expression; this differential effect is genuinely because of sleep versus novelty, as a brief period of sleep deprivation alone was shown to have minimal effects on memory consolidation; and (3) novelty and sleep are associated with differential patterns of up-regulation of immediate early genes in the hippocampus and the medial prefrontal cortex. We shall argue that these findings support the idea that the stabilization of memory traces within cell assemblies reflects a time-overlapping interaction of two interdependent processes—hippocampal and cortical consolidation.

### Hippocampal and Cortical Memory Consolidation

We begin by clarifying the distinction between hippocampal versus cortical consolidation on the one hand and cellular versus systems consolidation on the other. Cellular consolidation was initially identified by monitoring the impact of inhibitors of protein synthesis soon after training [43], with many later studies focusing on its expression in the hippocampus. Interestingly, consolidation within the hippocampus has been shown to be enhanced by postencoding novelty [13]. In contrast, systems consolidation was initially identified using perturbing interventions such as lesions via the phenomenon of retrograde amnesia [44], with more recent evidence indicating that this process may occur primarily during sleep [2]. Cortical consolidation likely involves both systems consolidation (interactions with the hippocampus) and cellular consolidation (stabilising synaptic changes within cortical networks). The hippocampal and cortical processes are generally held to act in sequence, with the hippocampal process setting the stage for the hippocampal–neocortical interactions that follow [26]. However, an alternative possibility supported by our data is that these two processes can occur in parallel and interact dynamically using both cellular and systems mechanisms, even if their respective time courses are not fully overlapping.

The novel approach here was to search for interactions between hippocampal and cortical consolidation in situations in which one or another competing memory trace might be rendered more dominant. This approach utilises the important concept of “dominance of the trace,” derived from studies of reconsolidation [30], which is particularly pertinent in situations involving potentially incompatible memory traces. With respect to the observations of Morris and Doyle [8], we can see that reversal training across days would have maximally enhanced cortical consolidation and lasting stabilisation, whereas within-day training would have enhanced a hippocampal trace with only minimal impact on cortical consolidation. The latter conditions would have been permissive for the spontaneous reversal during memory retention that was observed.

### Medial Prefrontal Cortex

One classic perspective on memory formation is that the hippocampus and cortex engage in “parallel encoding” [45–47] and that neocortical traces fade rapidly unless subject to a stabilisation signal from information retained within the hippocampus [48]. In contrast, the complementary learning systems theory initially suggested that rapid hippocampal encoding is sequentially followed by a slower neocortical “interleaving” process [26], a theory that has now been revised to recognise the possibility of “fast” consolidation [25]. The qPCR results showing similar up-regulation of IEGs in both structures within 30 min of memory encoding also support the “parallel encoding” concept, as they point to molecular events that are indirect markers of consolidation happening soon after memory encoding in both brain regions. IEG activation is triggered by memory encoding in both structures. Their interaction alters as a function of distinct behavioural manipulations such as postencoding novelty or sleep.

Others have proposed that the medial prefrontal cortex may not be a storage site for memory but, rather, responsible for memory integration and control [3,45–47]. Interestingly, the mPFC has to be active for reconsolidation to occur in the watermaze [48], and lesions in this area seem to especially affect memory retrieval under partial cueing conditions [49,50]. Here, sleep led to immediate early gene activation in the mPFC that, if the mPFC is for memory integration and behavioural control, could be associated with mPFC neural activity, leading to better integration of the two distinct experiences in the watermaze. This could explain our results in conditions with multiple experiences in the watermaze such as Pre-E and Int, in which the memory encoded prior to sleep was shown to dominate the control over behaviour. Interestingly, the opposite was the case in the Base condition, in which N + SD had the dominant control of behaviour. Independent of if the prefrontal cortex is the actual storage site or facilitates memory integration and control, in our case, plasticity processes occur in this region after learning and, more importantly, after sleep.

### Novelty

N + SD led to increased IEG expression in the hippocampus. According to the synaptic-tagging and capture- and clustered-plasticity models of consolidation [29,32,51], an increase of mRNA expression and translation caused by a novelty experience would enable newly synthesized, plasticity-related proteins to be captured by not only the initiating synapse (in this case, the one encoding the novel experience) but also by other synapses (in this case, the watermaze memory encoded 30 min earlier). The procedure we used of gentle handling of the animals and their frequent exposure to new objects in a novel environment likely sustained the relevant activation of protein synthesis over time, as evidenced by our IEG results showing sustained up-regulation over 6 h. Our interpretation is that these novelty-induced plasticity proteins would have triggered cellular consolidation of the watermaze memory in the hippocampus. This would have enabled the hippocampal trace to last long enough to be still present during a memory test conducted 24 h later in the case of our “Int” manipulation (Fig 2). With this trace retained and “active” in the hippocampus over this duration, the opportunity for training-associated updating would have prevailed [52]. The memory test would have enabled the animals to alter their hippocampal trace to indicate the escape platform was no longer available. Importantly, the trace consolidated in the cortex by sleep (see below) would have been relatively unaffected.

### Sleep

In contrast to novelty, the impact of sleep is that an initial learning-related up-regulation of relevant gene expression is sustained over time in the cortex (Fig 3), but this is in the context of a learning-independent decrease that becomes larger with time (Fig 4).

During sleep, activated synapses especially in the cortex are thought to be potentiated by intermittent replay events, putatively reflected in our sustained, learning-specific up-regulation of gene expression in the cortex [17,34,53–57]. However, sleep is also associated with a general time-dependent decrease in gene expression relative to home cage controls. Our study is the first to reveal extended time-dependent effects on IEG expression after longer periods of sleep, as earlier experiments have only investigated sleep for 1–2 h after learning [58,59]. This time-related decrease—possibly a “wash-out” of gene expression–related products—may be related to “downscaling” [34,60]. Downscaling is a type of cortical resetting process that is not yet well understood mechanistically [35,61–64]. The important idea is that within engram cells with potentiated synapses, synapses that are recently inactive are selectively downscaled, while the potentiated synapses of a new memory trace may be left intact [33,34,60].

In our case, downscaling was putatively reflected in the time-dependent decrease of gene expression that was learning independent. However, selectively after learning, a bimodality of cortical IEG expression was revealed as a sustained relative increase in gene expression during sleep, analogous to reported firing rate changes [35]. These bimodal changes are consistent with the concept of a selective strengthening of synapses involved in engrams—which most likely includes only a small percentage of neurons—and the general downscaling of all other synapses in mPFC during sleep, with a consequential enhancement of signal-to-noise ratio in the cortex as a signature of systems consolidation [16,17].

### Flipping the Seesaw

Preexposure to the environment enhanced the effectiveness of systems consolidation during sleep; in contrast, behavioural interference (a memory test) acted directly on the memory trace that had been enhanced soon after encoding by novelty with the effect of erasing it. The impact of 3 d of context preexposure would likely have created a network-level representation of the training context in the neocortex (and possibly in the hippocampus in parallel [65,66]). With that prior knowledge on the part of the animals, brief training was shown to be sufficient for rapid assimilation of information about the location of the hidden platform into the existing cortical representation, analogous to what happens in formal studies of knowledge assimilation into schemas [27,67]. In support, our behavioural analyses began with the demonstration of two search zones in animals with context preexposure who were trained to visit both escape platform locations in the pool; the cluster analysis quantitatively confirmed the existence of two separate clusters of memory traces at these two escape locations. Thus, while we present no evidence on the occurrence of rapid hippocampal independency as seen in Tse et al. [27], we did show a behavioural effect of the preexposure with the selective strengthening of the memory trace that was followed by sleep. In contrast, in the case of behavioural interference, as discussed above, the memory test acted directly on the memory trace that had been enhanced soon after encoding by novelty with the effect of erasing it. Here, the other memory whose consolidation was augmented by sleep was now the only one detectable.

### Potential Caveats

There are certain caveats to our approach. One is that the second session of learning in the watermaze might be thought to constitute “interference” trials for what was acquired in the first session of learning. Interestingly, our heat map and cluster analysis data establish that this did not occur. Instead, one or another trace “dominated” the control of behaviour, and the sequence in which they were learned had no statistically significant effect. Alterations to the stored memory representation of a hidden platform did occur when an interference test was conducted 24 h after training, but such a trial consisted of a long period of time swimming in the pool with multiple opportunities to learn that escape was no longer possible at a previously learned location. This new learning primarily affected what we suspect may be the hippocampal but not the cortical trace, but our data allows only that it was the memory trace that, in the original training the day before, was followed by novelty rather than by sleep. Second, dissociating a putative hippocampal and cortical trace is not straightforward; a complication is that spatial learning in the watermaze does not follow the usual temporal parameters of retrograde amnesia associated with posttraining hippocampal damage [68–70]. The reason is that the integrity of the hippocampus is required for the expression of spatial navigation. For example, “reminding” of a latent but inactive cortical spatial memory trace that is then expressed during a second retrieval trial requires the integrity of the hippocampus [68]. Accordingly, our group introduced an alternative way of investigating systems consolidation of watermaze learning in which the hippocampus functions normally at retrieval. Starting soon after training was completed, bilateral osmotic minipumps were used to infuse an AMPA receptor antagonist into the hippocampus for 7 d [71]. When the animals were tested 14 d after the end of training, the hippocampus was shown electrophysiologically to be working normally. Nonetheless, the impact of shutting down the hippocampus for 7 d was that spatial memory had been lost. On the basis of this and other earlier evidence [8,72], there are grounds for believing that the hippocampus and cortex interact normally for the consolidation of spatial learning in the watermaze. A third caveat concerns the possibility that the novelty manipulation has its effects because it is novelty and sleep deprivation. The procedural difficulty was that it is impossible to sustain a novelty manipulation for the same 6 h duration as the sleep manipulation we used without coupling it to sleep deprivation in order to ensure frequent access to novel stimuli. Further, because of the innate curiosity of the animals, novel stimuli act as natural agents of sleep deprivation. For this reason, we have throughout the results referred to our manipulation as “N + SD” and intend to investigate the potentially dissociable parameters of this protocol in future work. As an initial control experiment, we did repeat the interference experiment but this time with sleep deprivation by gentle handling but excluding novelty (see supplementary S11 Fig). This did abolish the differential effect of interference on the two memory traces, further indicating that our findings were primarily due to the effect of novelty and not sleep deprivation. Fourth, we recognise that the behavioural protocol differed for the qPCR study than for the earlier parts of the study, but as explained above (see Results), this was necessary for timing reasons and to avoid ambiguity.

### Conclusion

Marr proposed that sleep may be the ideal state for systems consolidation to occur [23,24]. However, the cyclical and unavoidable nature of this state in living animals makes exacting experimental designs to test causality very difficult to realise. Consequently, convincing tests of his ideas have been lacking. A growing body of data nonetheless points to the possibility that the hippocampal–neocortical interactions that reflect systems consolidation can occur more rapidly than others after Marr have generally considered [3,20,73]. An interventional approach inhibiting one or the other brain area during sleep or novelty would be ideal to test necessity or sufficiency, but the putative interdependence of the underlying processes limits the ability to achieve selective interventions. Nonetheless, our observations showing that hippocampal and cortical consolidation systems can interact offers further evidence for the dynamic nature of postencoding memory processing and their modulation by such factors as event-associated novelty, context preexposure, and postencoding sleep. The competitive memory study design sets the stage for future, more detailed investigations into the mechanisms of the hippocampal and cortical memory systems and their interactions.

## Methods

### Animals

The subjects were adult male Lister hooded rats (Charles River, United Kingdom [UK]), aged 8–10 wks at the start of experimentation and weighing ~250 g. They were housed in groups of four rats per cage. They had free access to food and water at all times and were kept on a delayed day–night cycle (behavioural experiments 12 p.m.–12 a.m. light on; qPCR experiments 10 a.m.–10 p.m. light on). After arrival, the animals adapted to the environment for at least a week and were handled across 3 d for at least 5 min per day before progressing to the watermaze habituation. A total of 305 rats were used (behavioural experiments: *n* = 160; qPCR experiments: *n* = 155).

### Ethics Statement

All procedures were compliant with national (Animals [Scientific Procedures] Act, 1986) and international (European Communities Council Directive of 24 November 1986[86/609/EEC]) legislation governing the maintenance of laboratory animals and their use in scientific experiments. We used the minimal number of rats for the necessary statistical power, with random assignment to groups, and there was minimal suffering associated with any of the experimental procedures. The experiments were approved by the UK home office under the project licence number 60/4566 and the Experimental Request Forms by the Edinburgh University division of the National Veterinary Service.

### Watermaze Habituation

The animals were habituated with a visual-cue version of the watermaze (diameter = 2 m) for 3 d prior to the main experimental day. During the four trials per day habituation, the rats had to find the submerged platform in the watermaze, indicated by a visual cue placed on top of the platform (diameter = 12 cm), while curtains surrounding the pool hid any extramaze cues. If the animals did not go to the platform within 120 s, they were guided to it by the experimenter (this occurred very rarely). After reaching the platform, the animals had to wait on the platform for 30 s before being picked up and the next trial began. All animals passed this habituation period with success, and with an average escape latency of <10 s on day 3. Thus, the animals were familiar with the procedure before spatial training was begun. To reduce further any experiment-associated stress, the animals were also habituated to the sleep cages (two times each for ~60 min) and the wake arena without any objects (15 min).

### Watermaze Training

We used two basic designs for watermaze training: a two-session design for the behavioural experiments and a one-session design for the qPCR experiments.

The main training day for the behavioural experiments consisted of two sessions in the watermaze separated by 7.5 h; each session was composed of four consecutive training blocks (each training block contained two trials, with a 15-s intertrial interval) to one platform location with randomized, counterbalanced, and varied starting positions (north, east, south, and west). If the animals did not reach the platform by 120 s, they were guided to the location. After each session, the animals underwent one of the two conditions (e.g., sleep, N + SD—see below) in a counterbalanced matter. Further, the platform position (northeast, southwest) was also counterbalanced across session and condition (see S1 Fig). Only animals that learned both platform locations successfully (block four escape latency < 55 s) were included in the zone analysis of the later probe trial. The probe trial was performed 7 d after the training day, and the animals were placed for 120 s in the watermaze without any platform present while their swim paths were tracked by automated software (Watermaze, Watermaze Software, Edinburgh, UK; [74]). Starting positions were counterbalanced and of equal distance to both platform locations (southwest, northeast). It should be noted that this software computes path parameters, including zone and dwell-time analyses, in a fully automatic manner—and is thus “blind” to the assignment of animals to groups and conditions.

For the qPCR experiments, a similar behavioural protocol was used for training, but with only one session to one platform location. A between-subject design was employed, with the single session of training (four blocks of two trials) to the single platform location followed by either N + SD or sleep before humane killing at varying intervals and RT-qPCR analysis of brain tissue.

In addition to the Base, Pre-E, and Int experiments of the behavioural study described in the main text, we performed an additional control experiment (S2 Fig). To investigate if the effects seen in the N + SD condition were indeed caused by novelty, we repeated the Int experiment using animals that had been sleep deprived with gentle handling rather than novelty exposure (Int-SD).

### Sleep and Novelty + Sleep Deprivation Procedures

In the sleep condition, the animals were kept in individual cages with video monitoring. Because of the high number of animals (>300), we were unable to perform implanted EEG recordings; instead, with the tracking data from the video monitoring of these animals, we calculated estimated sleep periods, defining sleep as at least >5 s with no movement [75]. We verified that total sleep time (M = 292.3 min ± 4.8) as well as length (M = 1.58 min ± 0.03) and number (M = 187.8 ± 3.1) of sleep bouts were similar to polysomnographic verified recordings.

Novelty with sleep deprivation was undertaken in individual compartments in an arena over a period of 6 h, with one experimenter assigned per four animals, their role being to monitor the rats and introduce novel objects as soon as the animal showed signs of tiredness (e.g., curling up). This procedure required considerable concentration by the experimenters and thus was done in shifts but was conducted in a conscientious manner (S4 Fig, left). Sleep deprivation (SD) without novelty (for the control studies) was performed in the home cages by gently tapping the cage or removing the cover as soon as the animal showed signs of tiredness (S4 Fig, right).

### Watermaze Analysis (Zone and Cluster)

We analysed the watermaze probe trial data in two ways: zone analysis of swim time and dwell time map–based cluster analysis. For each analysis, the data of 3–60 s of the probe trial was used to avoid the bias added by starting position.

#### Zone analysis

For the zone analysis, we extracted the swim time for each individual animal in the zone centred at the platform location (diameter = 86 cm). Chance level was determined by the pool area in the zone divided by the overall pool area.

#### Cluster analysis

Two-dimensional spatial dwell time maps were produced using the complete x and y coordinates of paths tracked from Watermaze that consisted of binned dwell time data, with each bin representing 1 pixel of the tracking area window. These maps were rotated by 180° for 50% of the animals (as necessary) and then averaged to give a mean dwell time map for all of the animals. This average dwell time map was then smoothed using a two-dimensional Gaussian kernel given by:

$$g\left(x\right)=exp\left(\frac{-x^{2}/\;2\sigma^{2}-y^{2}}{2\sigma^{2}}\right)$$

where σ was set to 2.5. This setting allowed smoothing of the dwell time map from a raw state without losing local dwell time features.

For further analyses to proceed, it was necessary to remove background noise and top threshold areas where the animals spent little time. The dwell time maps were normalized and bins removed where the dwell time was less than 50% of the maximum. We then isolated distinct regions of interest and removed regions with areas of less than 100 contiguous pixels. Custom MATLAB scripts were then used to determine the locations of local maxima in the remaining areas of the dwell time map.

Further MATLAB scripts were used to cluster these local maxima. We used the gap statistic method [37,76] to objectively quantify the optimal number of clusters in our local maxima, this being based on Euclidean distance values. Using the optimal number of clusters suggested by this method, local maxima were clustered using a hierarchical clustering method [77,78].

To quantify the characteristics of the resulting clusters, a number of measures were used. One was the average distance between every point and every other point in a cluster (PtoP) and another the average distance between every point in a cluster and the centroid of that cluster (PtoC); these two measures served to quantify the sparsity of a cluster. That is, the more dispersed the points are, the higher these two measures should be. For each cluster, we also calculated its convex hull (the smallest nonconcave polygon that can enclose all points in a cluster), the area of which was expressed as a percentage of the watermaze total area. For each cluster, the dwell time values found in the dwell time map at the location of each peak included was extracted for that cluster. This was used to determine the intensity of a cluster’s peak values. Further, we calculated the distance between the centre of the cluster and the actual platform location, indicating the accuracy of the memory. The importance of these measures lay in the prospect of identifying whether a spatial memory trace existed in circumstances due to the experimental design, where it may be dominated by another trace.

In order to determine if these clustering methods were biased towards a particular set of observations, we also analysed similarly composed sets of random data. For each experiment, a random set of local maxima locations were artificially created, equal in number to those observed in the real dataset and constrained within a circle of the same dimensions as our watermaze. These artificial maxima were clustered as described above, and the same measures were extracted for each. This process was repeated 100 times.

### RTqPCR Analysis

#### Experimental design

We performed qPCR measurements on brain tissue derived from two types of behavioural experiments: (1) encoding or consolidation, and (2) retrieval (see main Fig 3). With exception of the home cage controls (*n* = 5 and *n* = 16), all animals were trained with the one-session training protocol (see above).

The animals were culled either 30 min after encoding (*n* = 5) or at different intervals (2h, 4h, and 6h) after encoding with either sleep or N + SD in the intervening period (each *n* = 5). Further, we included a NoWM group (no exposure to the watermaze, also functions as home cage controls) directly at 30 min as well as both a NoWM and WM group after a 5 h interval with either Sleep or N + SD.

After the training day with either Sleep or N + SD, half the animals had a 24 h interference trial (similar to Base and Int experiments), and all animals were culled 30 min after the 7 d probe trial (each *n* = 16; S6 Fig). Immediately after extracting the brains, bilateral hippocampi and the medial prefrontal cortex were dissected and frozen with liquid nitrogen for later processing.

#### RT-qPCR

Briefly, samples were homogenized and RNA was obtained via phenol-chloroform extraction according to the manufacturers’ instruction. Next, cDNA was synthesized in vitro with use of random hexamers. Subsequently, a RT-qPCR and comparative Ct quantitation was performed in experimental duplicates for cFos, Arc, Zif268, and 18S ribosomal RNA as the internal control on a StepOnePlus (Applied Biosystems, Carlsbad, United States) PCR machine. Plates were counterbalanced and amplification thresholds set manually (StepOne Software Version 2.3, Life Technologies). The amplified product size was verified using gel electrophoresis and amplification checked for primer-dimer formation and nonefficient DNase treatment. Data was normalized to the internal control 18S (also known as Rn18s, coding for ribosomal RNA47), and subsequently “fold” and then “percentage change” to home cage control or other control was calculated. Percent (%) change was used for statistical analysis and graphical presentation because fold change cannot be used for statistics and percent change gives a more intuitive sense of effect sizes. Note that a fold change of 2 corresponds to +100%, whereas a fold change of 0.5 would be a –100% change. Thus, a fold change of 0.08 corresponds to –1,170% change. For comparison, both fold and percent change for the consolidation period are presented in S1–S3 Tables.

Further, to control for nonrelevant, confounding effects, we performed paired analysis between two experimental groups. For encoding and consolidation, we compared 30 min and 5 h after the watermaze encoding session animals that experienced the watermaze (WM) directly with animals that had not experienced the watermaze (NoWM). Thus, positive values on the *y*-axis show that animals consolidating a memory (WM) show higher gene expression than animals that are not consolidating a memory (NoWM), independent of what they are doing at the moment (sleep/N + SD). Additionally, to investigate the differences between sleep and N + SD animals at retrieval, we calculated percentage change (sleep > N + SD) on each plate and then averaged the data (see S7 Fig). Primers were: 18S left: atggttcctttggtcgctcg, right: ggcagacgttcgaatgggtc; Zif left: aacactttgtggcctgaacc, right: ggcagaggaagacgatgaag; cFos left: gagggagctgacagatacgc, right: tgggctgccaaaataaactc; Arc left: agctggaacctcatccacac, right: cagcagtggaactggtctca.

## Supporting Information

S1 Fig Counterbalanced design for the 2-session training day. Half the animals were trained in session 1 to a platform location in NW (top row) while the other half to SE (bottom row). Each group was subdivided and assigned to either Sleep or N+SD during the first consolidation window, after which each animal was trained to the other platform position and had subsequently the other experimental condition. Each behavior experiment (Base, Pre-E, Ext, Ext-SD, New P) used an n = 8 for each of the four sub-groups (total n = 32 per experiment). Note that in the data presentations of the main paper, the data derived from the top and bottom rows is pooled by rotating half of the data by 180°.
(TIF)

S2 Fig Supplemental experiment. The experimental design involved a group Ext, as described in the main text, that received an extinction trial (120s) 24h after the training day and followed by a 7d probe trial. Ext-SD used the same general design; however the sleep deprivation (SD) procedure involved gentle handling rather than novelty.
(TIF)

S3 Fig Sleep protocol. Left: animals in individual sleeping cages. Right: sleep periods in blue over the 6h consolidation period of an example animal. The animal switches continuously between sleep and wake periods.
(TIF)

S4 Fig Sleep deprivation with and without novelty. Procedures as described for N+SD (two pictures on the left) and SD (two pictures on the right).
(TIF)

S5 Fig Watermaze pictures. Watermaze pictures with extra-maze cues (left) and schematic (right). The pool had a radius of 1m, while the analysis zones centered on the platform locations had each a radius of 43cm.
(TIF)

S6 Fig Study design for the qPCR experiments investigating retrieval. Always half the animals were Sleep and N+SD for 6h post training.
(TIF)

S7 Fig qPCR plates. The qPCR data on each plate was analyzed in two ways. (A) For Encoding, Consolidation and Retrieval we calculated fold and then percentage change to home cage controls. That is, the blue triplicates for the sleep condition were compared with the white home-cage triplicates; likewise the orange N+SD triplicates. (B) Additional for Retrieval, we also calculated the relative fold-change of the two conditions (Sleep>N+SD) since these pairs controlled for additional effects e.g. swimming in the water maze.
(TIF)

S8 Fig Baseline experiment. Shown is the zone analysis of the Baseline or ‘primary’ experiment separated for the two sequences with sequence 1 Sleep followed by N+SD and sequence 2 N+SD followed by Sleep.
(TIF)

S9 Fig Primary experiment. Shown is the percent swim time in the zone for N+SD (y-axis) and for Sleep (x-axis) for each animal (within-subject experimental design). There was a significant negative correlation across all experiments (black line). The dotted line represents perfect anti-correlation (slope = -1.0). Note correlation was weakest for the Extinction condition for which the extinction caused an apparent loss of the memory representation for the N+SD platform location, causing the regression line to cross the y-axis (N+SD) on a lower value while the crossing at the x-axis (Sleep) becomes slight higher. *p <0.05, **p<0.01, ***p<0.001, ****p<0.0001.
(TIF)

S10 Fig Primary experiment. Shown are the latencies to reach the platform during the first and second session of the training day on all three experiments.
(TIF)

S11 Fig Supplement experiments. A. Control experiments (Base-SD and Int-SD) were run similarly to the Base and Int experiment described in the main text. For Base/Int-SD the animals were deprived of sleep during the consolidation window after encoding with gentle handling instead of novelty exposure to isolate its effect. B. There was no significant difference across the averages of the two zones, indicating normal swim behavior in all experiments. C. The zone analysis shows that only in the novelty experiments a differential effect of the conditions was seen across experiments. Novelty/gentle handling X condition X experiment interaction F = 3.7, df 1/116, p = 0.033 *p <0.05, **p<0.01 t-test to chance.
(TIF)

S12 Fig Visual illustration of the cluster analysis (Base experiment). (From left to right): Dwell Time Map, filtered Dwell Time Map, locations of peak activity (local maxima), peak locations displayed on Dwell Time Map. Automated cluster assignments of peak locations, point to center distances (PtoC), point to point distances (PtoP), cluster area (convex hull).
(TIF)

S13 Fig Cluster analysis. Presented are the different measures from the cluster analysis for the Base, Pre-E and Ext experiment. (Top row, left to right): Point to point distances (PtoP), point to center distance (PtoC), Average activity for the cluster peaks drawn from the dwell time maps; (Bottom row): Cluster size in % area of pool and pixel, distance from cluster centre to platform position. Both PtoP and Average Activity showed a significant condition (Sleep, N+SD) X experiment (Base, Pre-E) interaction (*p<0.05). Since only one cluster was present, Ext was not included in the statistical analysis.
(TIF)

S14 Fig RT-qPCR analysis. Shown as fold change (in contrast to % change of the main figure). HPC = hippocampus, mPFC = medial prefrontal cortex, N+SD = novelty with sleep deprivation, HC = home cage controls. Means +/- 1 SEM.
(TIF)

S15 Fig Retrieval-associated IEG expression. A direct comparison of N+SD with Sleep at retrieval is displayed with positive values reflecting higher gene expression in Sleep and negative values higher gene expression in N+SD. A gene x brain area interaction was seen (F = 4.4, df 2/60, p<0.03, with post-hoc linear contrast p<0.03), with Sleep showing higher cFos expression in the HPC but higher Arc and Zif-268 expression in the mPFC, with the opposite pattern for N+SD.
(TIF)

S16 Fig Retrieval-associated IEG expression. Left panel: At retrieval in comparison to HC, HPC showed higher changes in IEG expression than mPFC (F = 14.7, df 1/60, p<0.001) and, contrasting to encoding (i.e. left panel vs Fig 3B), a significant gene x trial type effect was seen (F = 3.9, df 1.28/85.9, p<0.05; Greenhouse-Geisser correction). Right panel: IEG expression is plotted as percentage change relative to home-cage control, and separated into groups of animals that either had (Exp A, blue) or did not have (Exp B) a retrieval trial 24 hr after training as well as at the 7d test just prior to culling. ** p<0.01, ***p<0.001.
(TIF)

S17 Fig Time spent sleeping and gene expression. The correlations between IEG expression measured after the consolidation period in the Sleep condition (all three 2, 4, 6h) and the amount of time spend sleeping (as measured by motion analysis). There were no significant effects in the HPC, but all three genes showed a significant negative correlation between the amount of time asleep (total sleep time, TST) and gene expression in comparison to home cage controls. ** p<0.01, ***p<0.001.
(TIF)

S18 Fig Paths. Shown are example paths of individual animals in the Base, Pre-E and Int experiments.
(TIF)

S1 Table Percent change to HC. (PDF)

S2 Table Fold change to HC. (PDF)

S3 Table qPCR analysis consolidation period. ANOVA with within-subject factors gene, brain area (BA) and between-subject factors condition (Sleep/N+SD, con) and time (2,4,6h).
(PDF)

S4 Table Swim time zone analysis. Shown are the values for each animal for the Sleep and N+SD zone. Chance level 17.6%.
(PDF)

S1 Text Supplemental results. (DOCX)

S1 Data Raw data. (XLSX)

## Abbreviations

HCC: home cage control
HPC: hippocampus
IEG: immediate early gene
mPFC: medial prefrontal cortex
N + SD: novelty + sleep deprivation
NoWM: no exposure to the watermaze
WM: watermaze
