# Supplementary figures and images for: The Yin and Yang of Memory Consolidation: Hippocampal and Neocortical
Source: PLoS Biol. 2017 Jan 13;15(1):e2000531. doi: 10.1371/journal.pbio.2000531 (PMC5234779; doi:10.1371/journal.pbio.2000531)

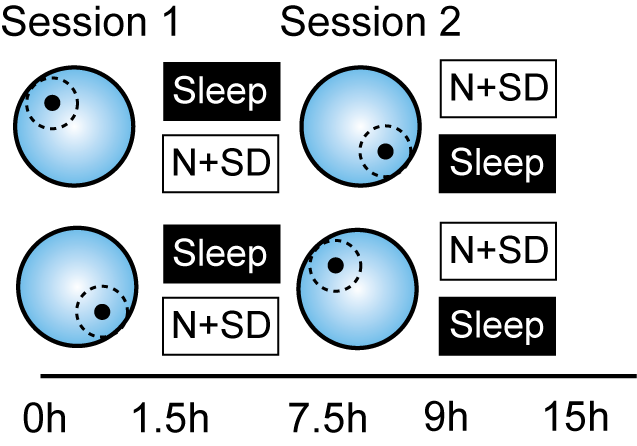

Supplement: S1 Fig — Half the animals were trained in session 1 to a platform location in NW (top row) while the other half to SE (bottom row). Each group was subdivided and assigned to either Sleep or N+SD during the first consolidation window, after which each animal was trained to the other platform position and had subsequently the other experimental condition. Each behavior experiment (Base, Pre-E, Ext, Ext-SD, New P) used an n = 8 for each of the four sub-groups (total n = 32 per experiment). Note that in the data presentations of the main paper, the data derived from the top and bottom rows is pooled by rotating half of the data by 180°. (TIF) [file pbio.2000531.s001.tif]

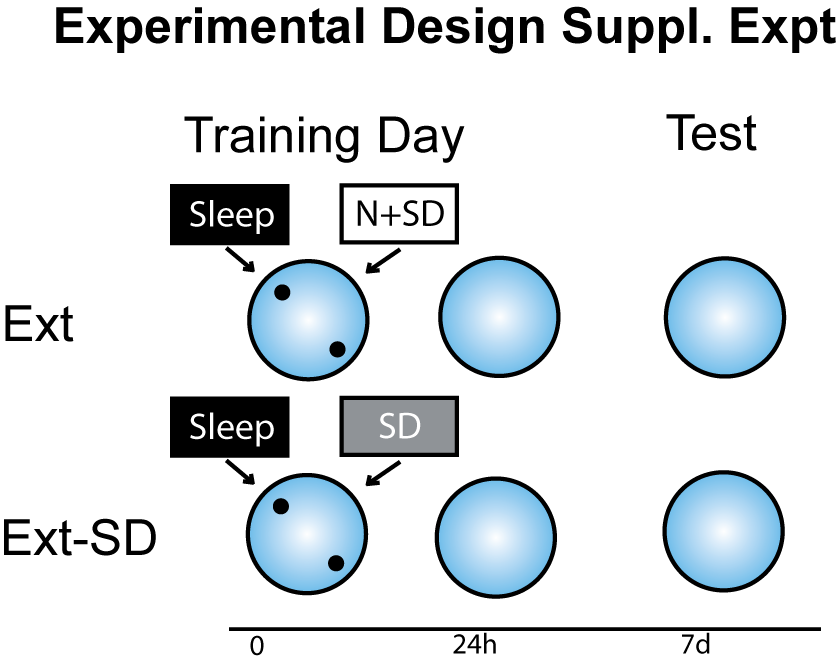

Supplement: S2 Fig — The experimental design involved a group Ext, as described in the main text, that received an extinction trial (120s) 24h after the training day and followed by a 7d probe trial. Ext-SD used the same general design; however the sleep deprivation (SD) procedure involved gentle handling rather than novelty. (TIF) [file pbio.2000531.s002.tif]

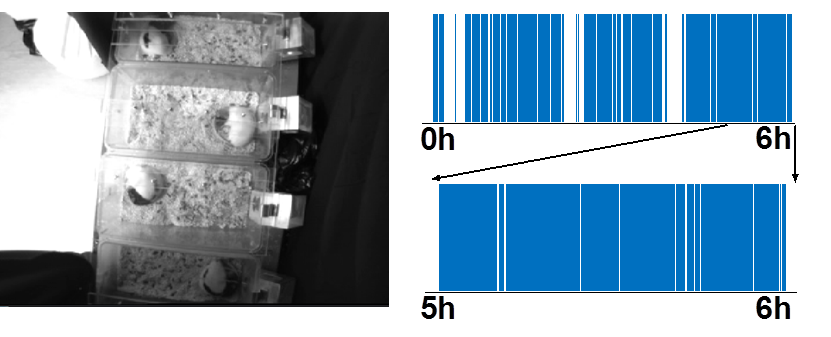

Supplement: S3 Fig — Left: animals in individual sleeping cages. Right: sleep periods in blue over the 6h consolidation period of an example animal. The animal switches continuously between sleep and wake periods. (TIF) [file pbio.2000531.s003.tif]

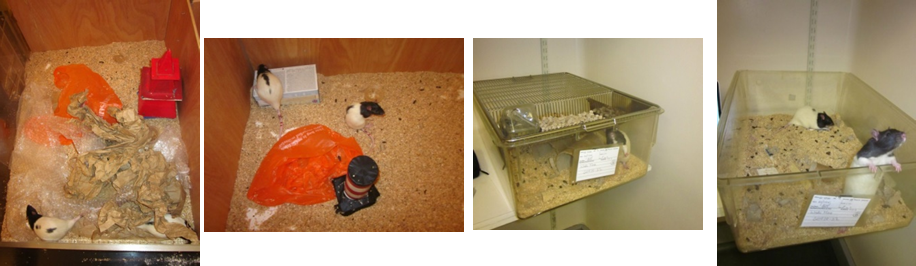

Supplement: S4 Fig — Procedures as described for N+SD (two pictures on the left) and SD (two pictures on the right). (TIF) [file pbio.2000531.s004.tif]

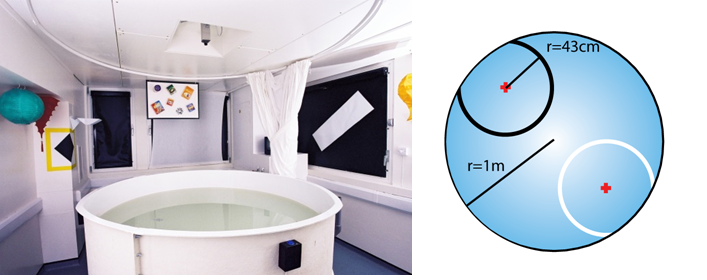

Supplement: S5 Fig — Watermaze pictures with extra-maze cues (left) and schematic (right). The pool had a radius of 1m, while the analysis zones centered on the platform locations had each a radius of 43cm. (TIF) [file pbio.2000531.s005.tif]

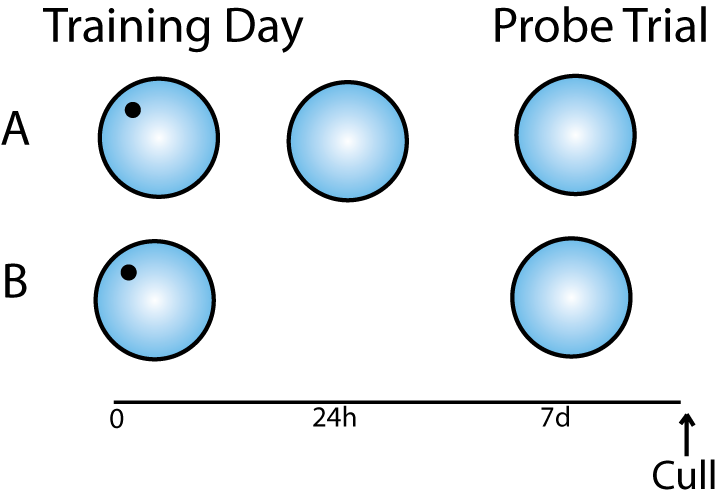

Supplement: S6 Fig — Always half the animals were Sleep and N+SD for 6h post training. (TIF) [file pbio.2000531.s006.tif]

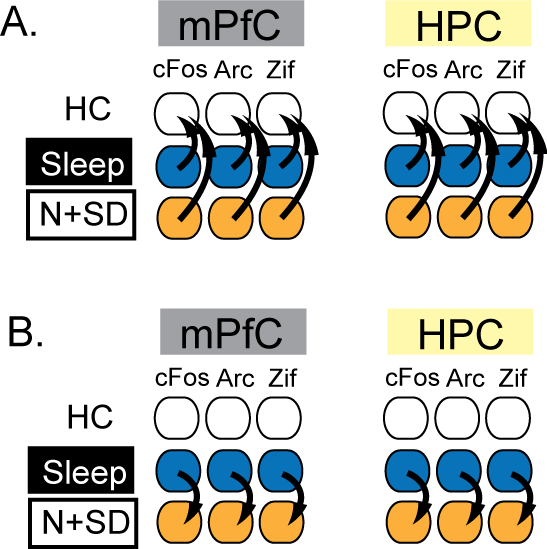

Supplement: S7 Fig — The qPCR data on each plate was analyzed in two ways. (A) For Encoding, Consolidation and Retrieval we calculated fold and then percentage change to home cage controls. That is, the blue triplicates for the sleep condition were compared with the white home-cage triplicates; likewise the orange N+SD triplicates. (B) Additional for Retrieval, we also calculated the relative fold-change of the two conditions (Sleep>N+SD) since these pairs controlled for additional effects e.g. swimming in the water maze. (TIF) [file pbio.2000531.s007.tif]

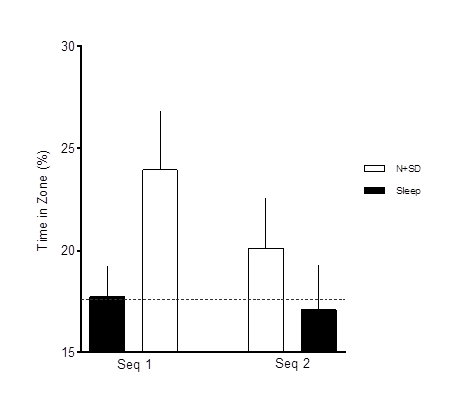

Supplement: S8 Fig — Shown is the zone analysis of the Baseline or ‘primary’ experiment separated for the two sequences with sequence 1 Sleep followed by N+SD and sequence 2 N+SD followed by Sleep. (TIF) [file pbio.2000531.s008.tif]

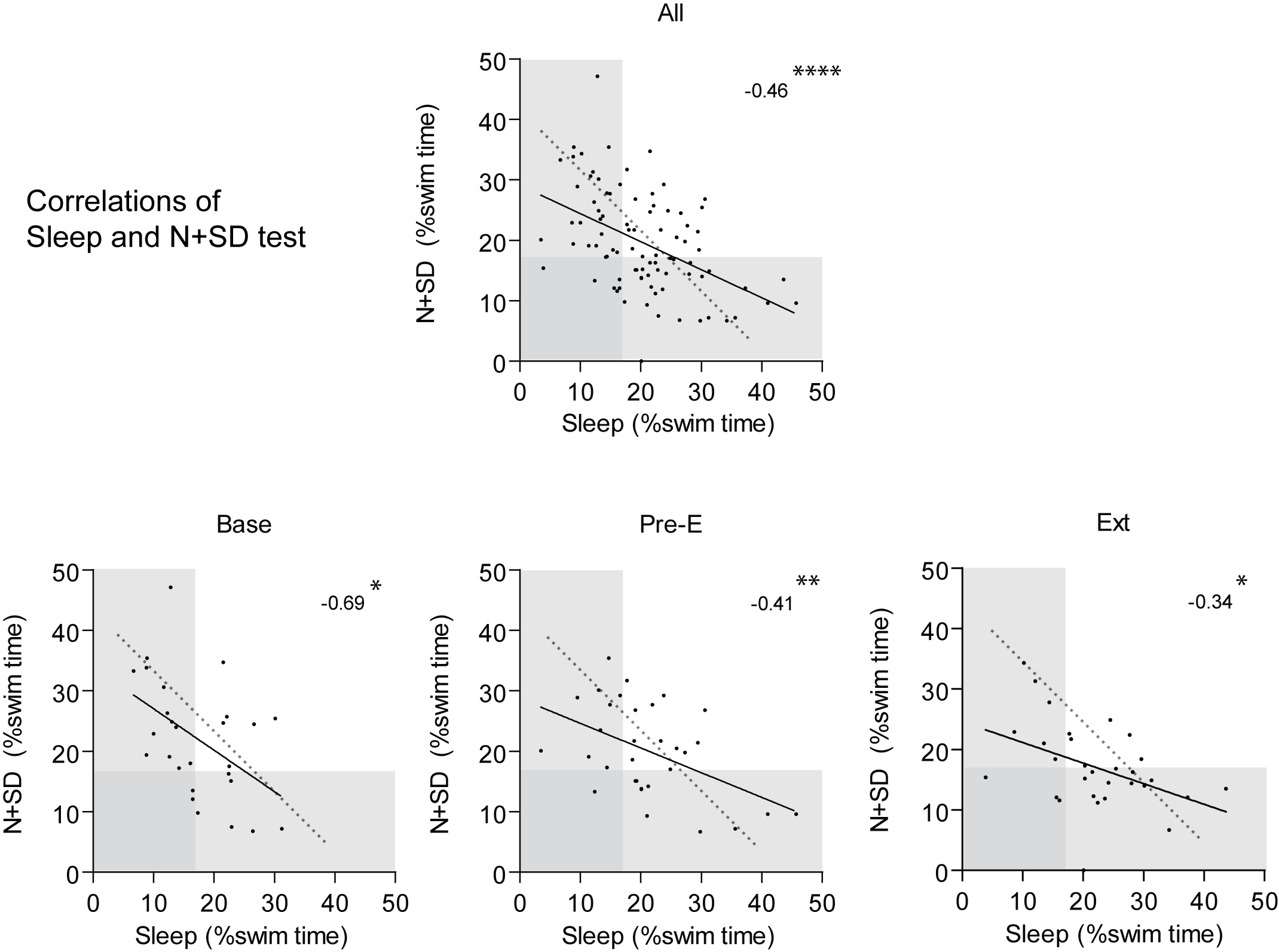

Supplement: S9 Fig — Shown is the percent swim time in the zone for N+SD (y-axis) and for Sleep (x-axis) for each animal (within-subject experimental design). There was a significant negative correlation across all experiments (black line). The dotted line represents perfect anti-correlation (slope = -1.0). Note correlation was weakest for the Extinction condition for which the extinction caused an apparent loss of the memory representation for the N+SD platform location, causing the regression line to cross the y-axis (N+SD) on a lower value while the crossing at the x-axis (Sleep) becomes slight higher. *p <0.05, **p<0.01, ***p<0.001, ****p<0.0001. (TIF) [file pbio.2000531.s009.tif]

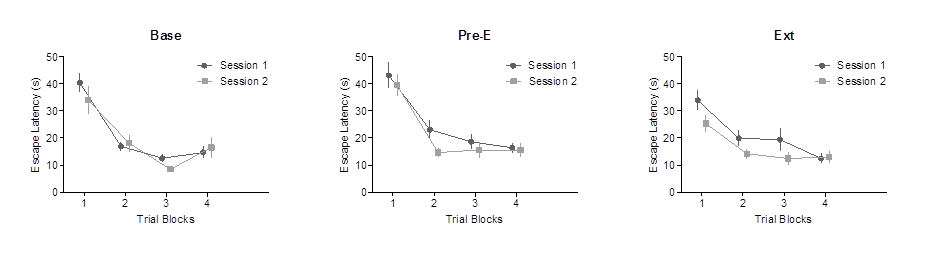

Supplement: S10 Fig — Shown are the latencies to reach the platform during the first and second session of the training day on all three experiments. (TIF) [file pbio.2000531.s010.tif]

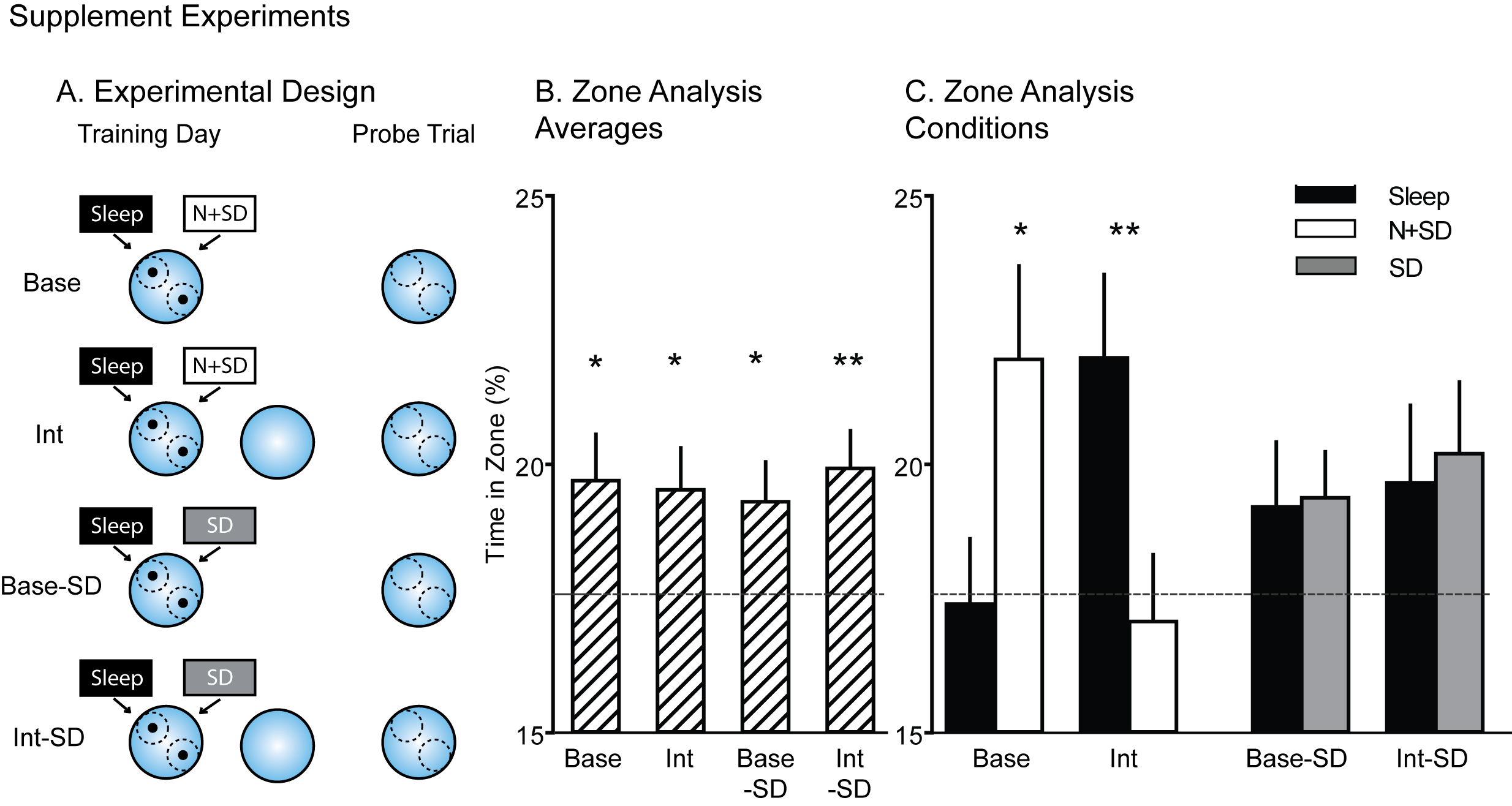

Supplement: S11 Fig — A. Control experiments (Base-SD and Int-SD) were run similarly to the Base and Int experiment described in the main text. For Base/Int-SD the animals were deprived of sleep during the consolidation window after encoding with gentle handling instead of novelty exposure to isolate its effect. B. There was no significant difference across the averages of the two zones, indicating normal swim behavior in all experiments. C. The zone analysis shows that only in the novelty experiments a differential effect of the conditions was seen across experiments. Novelty/gentle handling X condition X experiment interaction F = 3.7, df 1/116, p = 0.033 *p <0.05, **p<0.01 t-test to chance. (TIF) [file pbio.2000531.s011.tif]

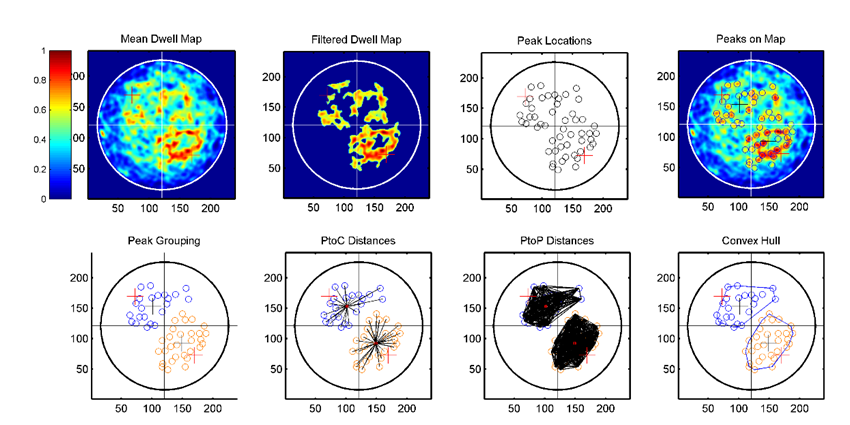

Supplement: S12 Fig — (From left to right): Dwell Time Map, filtered Dwell Time Map, locations of peak activity (local maxima), peak locations displayed on Dwell Time Map. Automated cluster assignments of peak locations, point to center distances (PtoC), point to point distances (PtoP), cluster area (convex hull). (TIF) [file pbio.2000531.s012.tif]

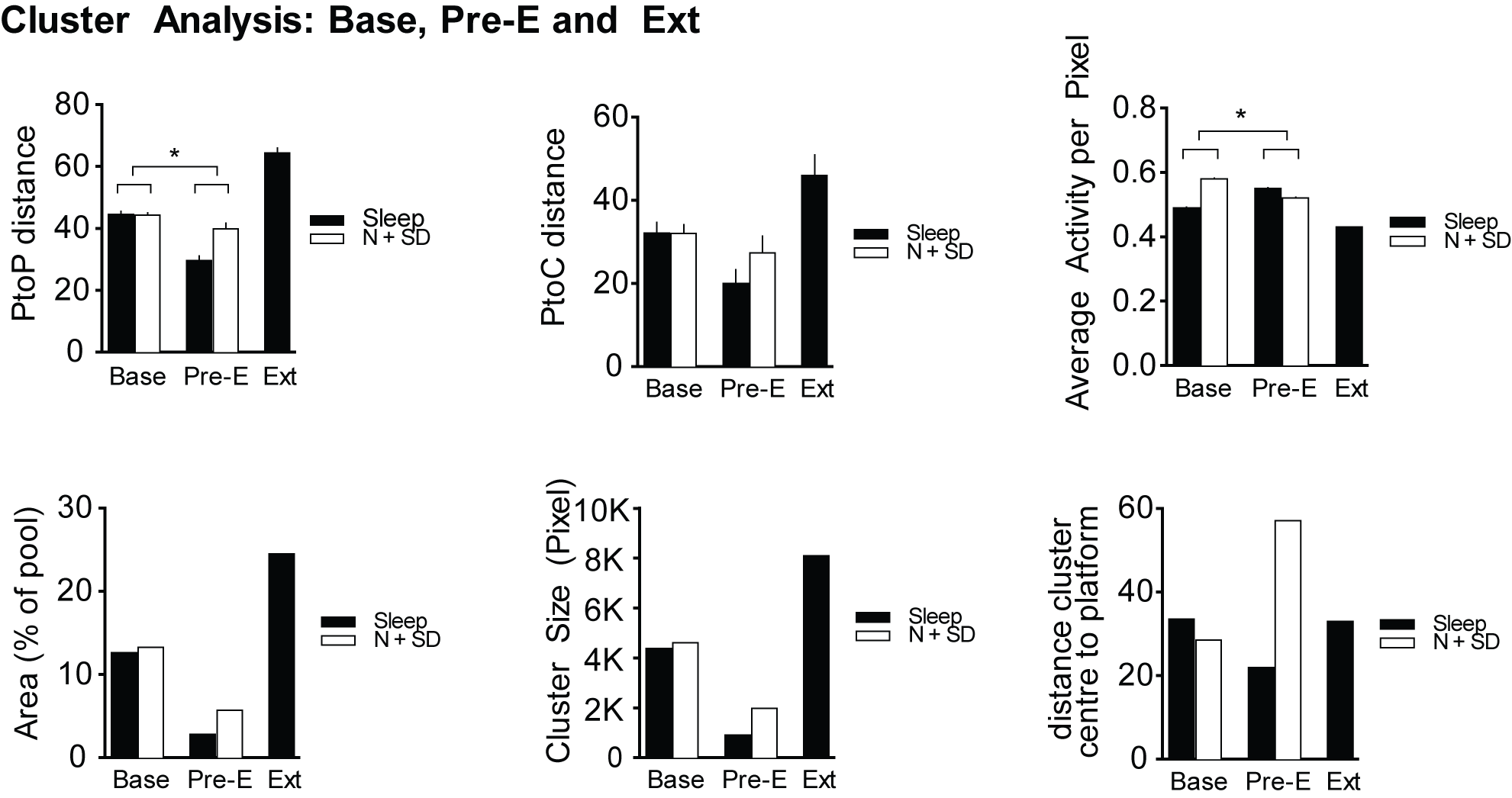

Supplement: S13 Fig — Presented are the different measures from the cluster analysis for the Base, Pre-E and Ext experiment. (Top row, left to right): Point to point distances (PtoP), point to center distance (PtoC), Average activity for the cluster peaks drawn from the dwell time maps; (Bottom row): Cluster size in % area of pool and pixel, distance from cluster centre to platform position. Both PtoP and Average Activity showed a significant condition (Sleep, N+SD) X experiment (Base, Pre-E) interaction (*p<0.05). Since only one cluster was present, Ext was not included in the statistical analysis. (TIF) [file pbio.2000531.s013.tif]

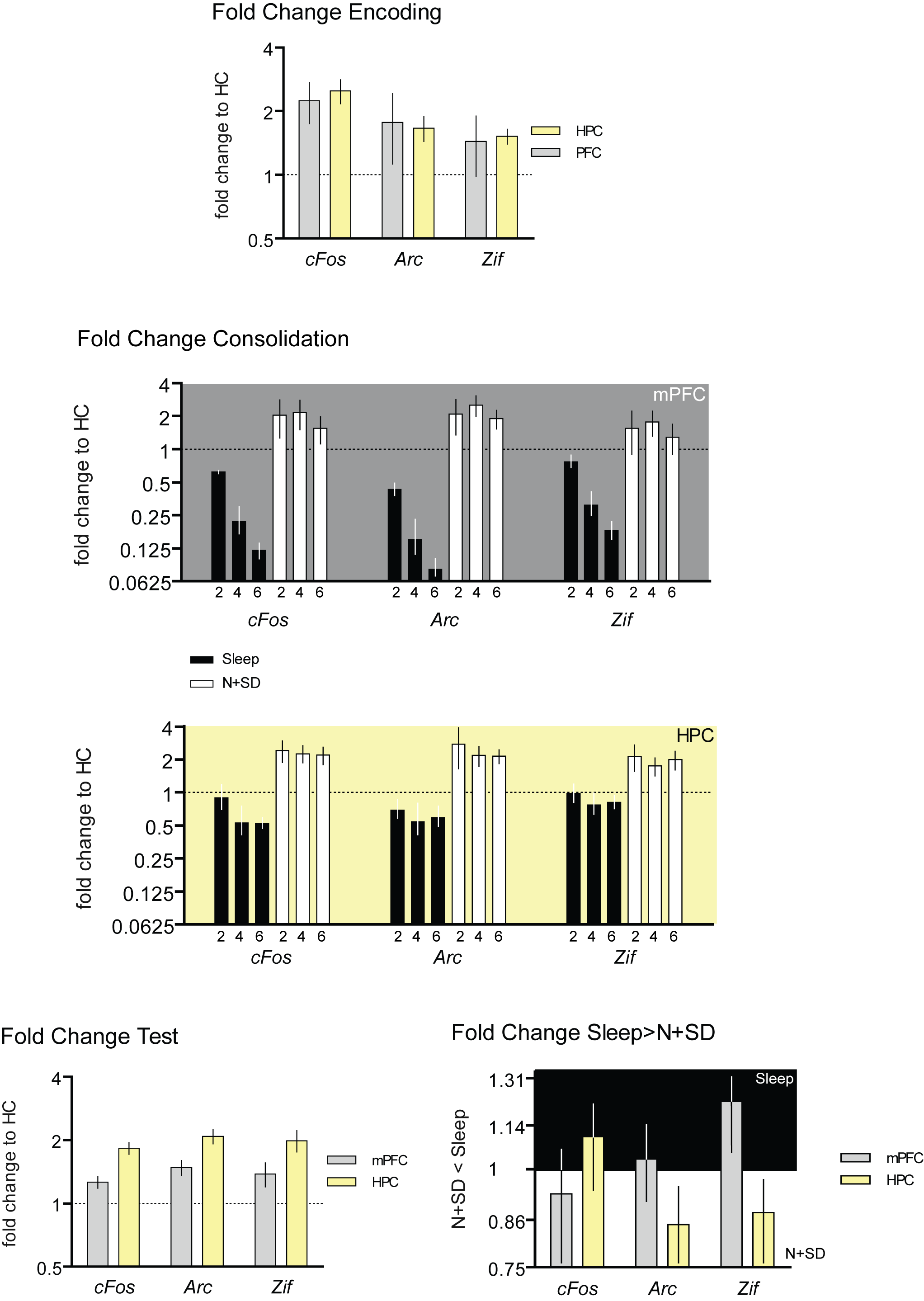

Supplement: S14 Fig — Shown as fold change (in contrast to % change of the main figure). HPC = hippocampus, mPFC = medial prefrontal cortex, N+SD = novelty with sleep deprivation, HC = home cage controls. Means +/- 1 SEM. (TIF) [file pbio.2000531.s014.tif]

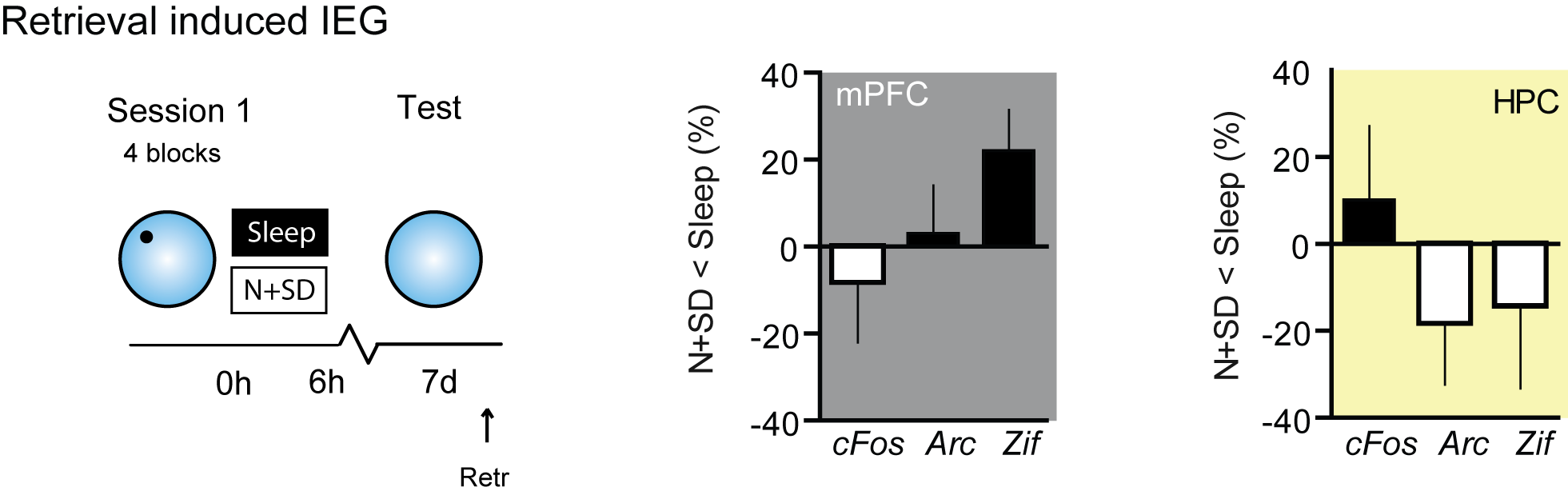

Supplement: S15 Fig — A direct comparison of N+SD with Sleep at retrieval is displayed with positive values reflecting higher gene expression in Sleep and negative values higher gene expression in N+SD. A gene x brain area interaction was seen (F = 4.4, df 2/60, p<0.03, with post-hoc linear contrast p<0.03), with Sleep showing higher cFos expression in the HPC but higher Arc and Zif-268 expression in the mPFC, with the opposite pattern for N+SD. (TIF) [file pbio.2000531.s015.tif]

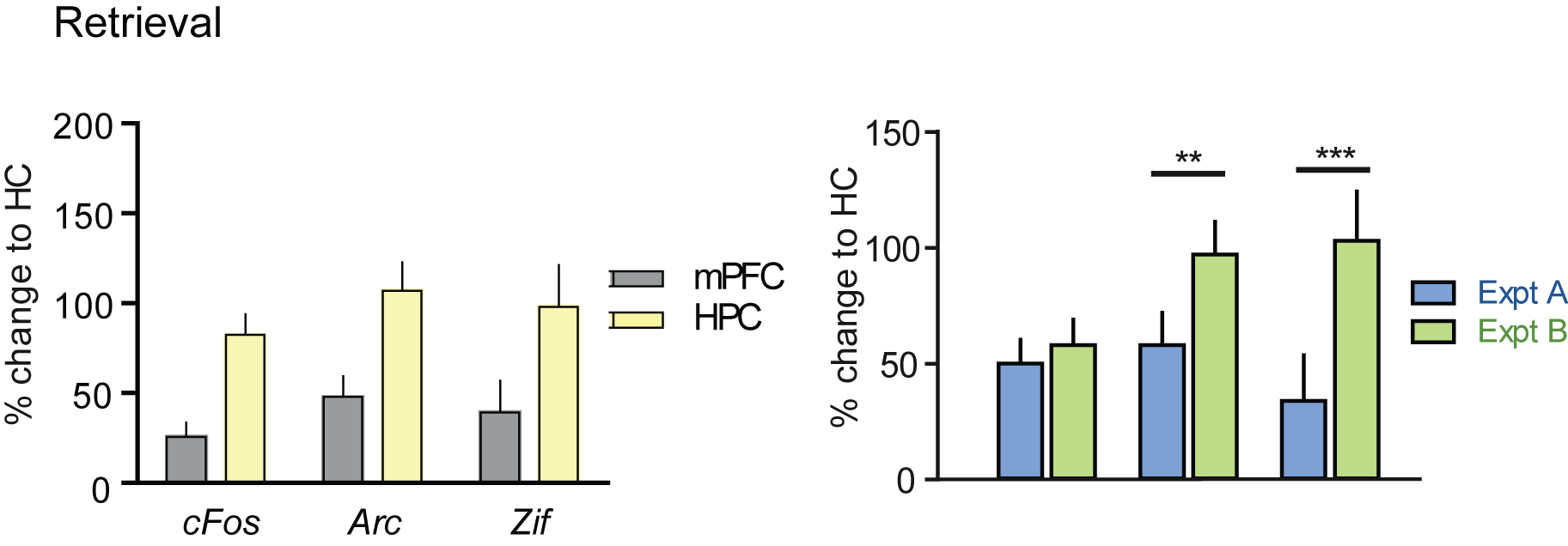

Supplement: S16 Fig — Left panel: At retrieval in comparison to HC, HPC showed higher changes in IEG expression than mPFC (F = 14.7, df 1/60, p<0.001) and, contrasting to encoding (i.e. left panel vs Fig 3B), a significant gene x trial type effect was seen (F = 3.9, df 1.28/85.9, p<0.05; Greenhouse-Geisser correction). Right panel: IEG expression is plotted as percentage change relative to home-cage control, and separated into groups of animals that either had (Exp A, blue) or did not have (Exp B) a retrieval trial 24 hr after training as well as at the 7d test just prior to culling. ** p<0.01, ***p<0.001. (TIF) [file pbio.2000531.s016.tif]

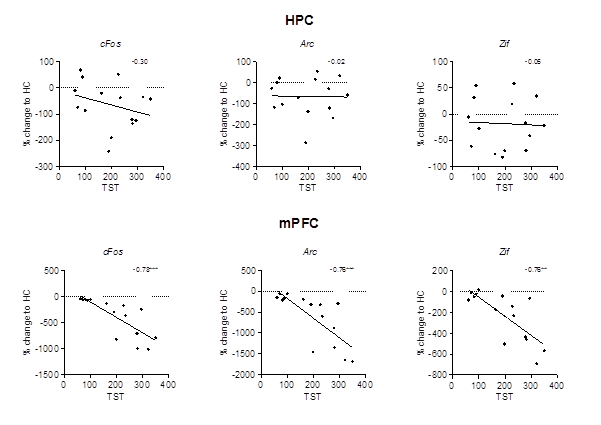

Supplement: S17 Fig — The correlations between IEG expression measured after the consolidation period in the Sleep condition (all three 2, 4, 6h) and the amount of time spend sleeping (as measured by motion analysis). There were no significant effects in the HPC, but all three genes showed a significant negative correlation between the amount of time asleep (total sleep time, TST) and gene expression in comparison to home cage controls. ** p<0.01, ***p<0.001. (TIF) [file pbio.2000531.s017.tif]

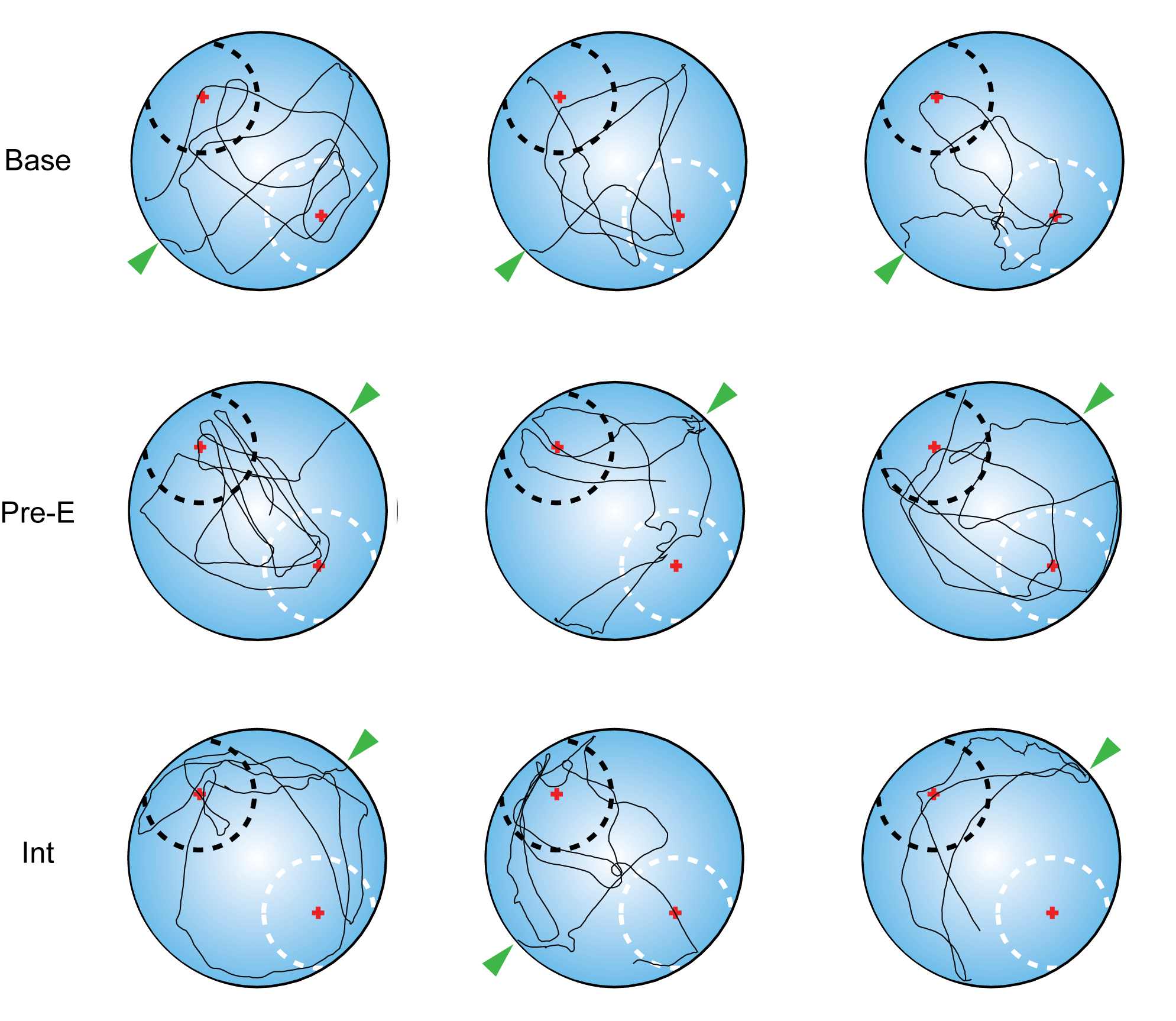

Supplement: S18 Fig — Shown are example paths of individual animals in the Base, Pre-E and Int experiments. (TIF) [file pbio.2000531.s018.tif]
