# Supplementary material for: The Yin and Yang of Memory Consolidation: Hippocampal and Neocortical
Source: PLoS Biol. 2017 Jan 13;15(1):e2000531. doi: 10.1371/journal.pbio.2000531 (PMC5234779; doi:10.1371/journal.pbio.2000531)
Supplement: S1 Table — (PDF) [file pbio.2000531.s019.pdf]

		cFos		*Arc*		*Zif*	
		M	SEM	M	SEM	M	SEM
enc	HPC	148.98	31.90	65.61	22.23	52.22	11.95
	mPFC	124.28	49.78	77.34	64.85	44.08	45.67
Sleep2h	HPC	-12.33	30.69	-44.64	27.95	-1.53	20.72
	mPFC	-61.95	4.82	-131.92	29.86	-30.32	16.77
N+SD2h	HPC	141.10	54.37	175.70	111.81	113.48	57.61
	mPFC	104.17	76.77	108.94	73.95	56.11	66.50
Sleep4h	HPC	-87.84	55.17	-84.98	60.54	-30.41	28.96
	mPFC	-357.77	121.92	-578.60	230.10	-218.27	78.08
N+SD4h	HPC	125.68	40.81	117.22	44.92	73.62	31.69
	mPFC	115.41	64.56	152.77	53.74	76.86	44.97
Seep6h	HPC	-92.00	22.00	-69.20	35.31	-23.20	17.14
	mPFC	-752.57	137.93	-1170.48	261.05	-444.32	105.01
N+SD6h	HPC	118.59	39.84	114.21	30.71	98.57	38.05
	mPFC	55.15	42.81	90.19	37.10	29.35	39.68
