# Supplementary material for: The Yin and Yang of Memory Consolidation: Hippocampal and Neocortical
Source: PLoS Biol. 2017 Jan 13;15(1):e2000531. doi: 10.1371/journal.pbio.2000531 (PMC5234779; doi:10.1371/journal.pbio.2000531)
Supplement: S2 Table — (PDF) [file pbio.2000531.s020.pdf]

		cFos	*Arc*	*Zif*
		M	M	M
enc	HPC	2.49	1.66	1.52
	mPFC	2.24	1.77	1.44
Sleep2h	HPC	0.89	0.69	0.98
	mPFC	0.62	0.43	0.77
N+SD2h	HPC	2.41	2.76	2.13
	mPFC	2.04	2.09	1.56
Sleep4h	HPC	0.53	0.54	0.77
	mPFC	0.22	0.15	0.31
N+SD4h	HPC	2.26	2.17	1.74
	mPFC	2.15	2.53	1.77
Seep6h	HPC	0.52	0.59	0.81
	mPFC	0.12	0.08	0.18
N+SD6h	HPC	2.19	2.14	1.99
	mPFC	1.55	1.90	1.29
