# Supplementary material for: The Yin and Yang of Memory Consolidation: Hippocampal and Neocortical
Source: PLoS Biol. 2017 Jan 13;15(1):e2000531. doi: 10.1371/journal.pbio.2000531 (PMC5234779; doi:10.1371/journal.pbio.2000531)
Supplement: S3 Table — ANOVA with within-subject factors gene, brain area (BA) and between-subject factors condition (Sleep/N+SD, con) and time (2,4,6h). (PDF) [file pbio.2000531.s021.pdf]

Test	F	Sig.
BA	18.654	.000
BA X Con	13.126	.001
BA X Time	4.644	.020
BA X Con X Time	4.440	.023
Gene	13.418	.000
Gene X Con	34.265	.000
Gene X Time	4.295	.011
Gene X Con X Time	3.321	.030
BA X Gene	12.515	.001
BA X Gene X Con	16.383	.000
BA X Gene X Time	3.173	.050
BA X Gene X Con X Time	4.652	.014

Test	F	Sig.
Con	69.849	.000
Time	8.678	.001
Con X Time	6.147	.007
