# Supplementary material for: The Yin and Yang of Memory Consolidation: Hippocampal and Neocortical
Source: PLoS Biol. 2017 Jan 13;15(1):e2000531. doi: 10.1371/journal.pbio.2000531 (PMC5234779; doi:10.1371/journal.pbio.2000531)
Supplement: S4 Table — Shown are the values for each animal for the Sleep and N+SD zone. Chance level 17.6%. (PDF) [file pbio.2000531.s022.pdf]

	Base		Pre-E		Int	
Nr	Sleep	N+SD	Sleep	N+SD	Sleep	N+SD
1	22.9	7.5	3.5	20.1	8.6	22.9
2	12.6	19.1	25.9	20.5	28.2	16.3
3	6.7	33.3	13.3	23.5	31.3	14.9
4	16.5	12.1	20.1	13.7	21.5	16.3
5	12.3	26.3	45.7	9.6	17.7	22.6
6	13.0	24.9	17.7	31.7	29.6	18.4
7	30.1	25.4	21.9	27.7	24.2	14.5
8	11.7	30.6	29.8	6.7	18.0	21.7
9	10.0	22.9	23.8	29.2	25.4	16.8
10	21.5	34.7	13.0	30.1	37.3	12.1
11	22.4	16.3	21.0	9.3	20.1	0.0
12	17.3	9.8	19.3	15.1	21.7	12.3
13	16.1	18.0	29.4	21.4	10.2	34.3
14	22.5	17.5	18.9	21.7	43.6	13.5
15	12.8	47.1	27.3	19.8	13.5	21.0
16	14.2	17.2	19.1	15.1	30.1	14.0
17	26.4	6.8	16.6	29.2	3.9	15.4
18	22.8	15.1	23.3	21.7	14.4	27.8
19	8.9	35.4	41.0	9.6	20.3	17.3
20	31.2	7.2	14.4	17.3	20.3	15.2
21	8.8	19.4	11.4	19.1	34.2	6.7
22	13.7	24.0	24.9	17.0	24.5	24.9
23	26.6	24.5	18.6	18.6	23.6	11.9
24	16.5	13.5	19.1	26.8	15.4	18.4
25	8.8	33.8	20.1	13.8	16.1	11.6
26	22.1	25.7	30.6	26.8	27.7	22.4
27	21.5	24.7	14.7	35.4	12.1	31.3
28			12.4	13.3	22.4	11.2
29			9.5	28.9	28.0	14.4
30			21.2	14.2	15.6	12.1
31			35.6	7.2		
32			14.9	27.7		
