# Supplementary material for: The Yin and Yang of Memory Consolidation: Hippocampal and Neocortical
Source: PLoS Biol. 2017 Jan 13;15(1):e2000531. doi: 10.1371/journal.pbio.2000531 (PMC5234779; doi:10.1371/journal.pbio.2000531)
Supplement: S1 Text — (DOCX) [file pbio.2000531.s023.docx]

# The yin and yang of memory consolidation: hippocampal and neocortical

## Supplement Materials

Lisa Genzel*, Janine I Rossato, Justin Jacobse, Roddy M Grieves, Patrick A Spooner, Francesco P Battaglia, Guillen Fernández^,^ Richard GM Morris

*corresponding author

**Statistical Analysis:**

GLM models were used, calculating ANOVAs with different factors.

Escape Latency: between-subject factors: sequence (1^st^ Sleep, 2^nd^ N+SD, 1^st^ N+SD, 2^nd^ Sleep); within-subject factors: session (1/2), trial block (1,2,3,4)

Zone Analysis: between-subject factors: experiment (Base, Pre-E, Ext), sequence (1^st^ Sleep, 2^nd^ N+SD, 1^st^ N+SD, 2^nd^ Sleep); within-subject factors: condition (Sleep, N+SD),

qPCR Analysis encoding: within-subject factors: brain area (HPC, mPFC), gene (*cFos, Arc, Zif*)

qPCR Analysis consolidation: between-subject factors: condition (Sleep, N+SD), time (2,4,6h); within-subject factors: brain area (HPC, mPFC), gene (*cFos, Arc, Zif*)

qPCR Analysis retrieval and encoding: between-subject factors: trial type (encoding, retrieval); within-subject factors: brain area (HPC, mPFC), gene (*cFos, Arc, Zif*)

qPCR Analysis retrieval to HC: between-subject factors: experiment (with, without 24h extinction trial), condition (Sleep, N+SD); within-subject factors: brain area (HPC, mPFC), gene (*cFos, Arc, Zif*)

qPCR Analysis retrieval pairs: between-subject factors: experiment (with, without 24h extinction trial); within-subject factors: brain area (HPC, mPFC), gene (*cFos, Arc, Zif*)

**Additional Control Experiment (Base and Int-SD)**

In addition to the behavioral experiments described in the main text, we ran a control experiments addressing the role of trace-dominance with respect to the impact of novelty.

For the Base and Int+SD experiment, we used a similar design as for the Base and Int experiment. These studies had shown the largest differential affect between Sleep and N+SD, but we wanted to check that the effect seen was not just due to the absence of sleep during the 6 hr N+SD experience. The experimental protocol was repeated, excepting the replacement of the N+SD condition by sleep deprivation accompanied by gentle handling (SD). The average performance of the two groups (Sleep and SD) was above chance at 7d in each experiment; however, in contrast to what had been observed in the original experiments, these two groups did not differ (see Figure S11). This somewhat paradoxical finding implies that the strengthening associated with novelty during N+SD results in one spatial memory becoming the dominant trace, and it therefore most affected by the interference procedure occurring soon after.

**Results Cluster Analysis**

*Base Experiment*

The number of local maxima detected was 51 but the optimal number of clusters for these maxima was found to be only 2 (22 peaks and 29 peaks respectively). After clustering, one cluster was found to fall predominantly in the NW quadrant (15 peaks or 68.18%), with the centroid of this cluster located close to the NW platform location. The second cluster fell predominantly in the SE quadrant (20 peaks or 68.97%) with its centroid closest to the SE platform position. This is a striking result of the cluster analysis.

Our shuffled data reveal that on average, the number of peaks would be expected on average to form two distinct clusters only 3% of the time, thus our observations deviate significantly from what would be expected by chance (a p = .03 with 30 occurrences in 1000 iterations).

The two clusters did not differ in the distances between their points [t(49) < 1, p > .99] or in the distances between points and the cluster centres [t(1272) = < 1, p > .92] nor was there a large difference in their convex hulls (12.64 and 13.29 respectively). These results suggest that the two groups are of a similar size and density.

*Pre-E Experiment*

The number of local maxima detected was 23, the optimal number of clusters for these maxima was found to be 2 (11 peaks and 12 peaks respectively). After clustering, one cluster was found to fall predominantly in the NW quadrant (11 peaks or 100%), the centroid of this cluster was also located closest to the NW platform location. The second cluster fell predominantly in the SW quadrant (7 peaks or 58.33%) and its centroid was closest to the SW platform location.

Our shuffled data reveal that, on average, this number of peaks would be expected to form two distinct clusters 0% of the time, meaning that our observation deviates significantly from what would be expected by chance (a p < .001 with 0 occurrences in 1000 iterations).

The two clusters did not differ in the distances between their points [t(21) = -1.39, p > .17] but they did differ in the distances between points and the cluster centres [t(240) = -4.19, p < .001]. There was also a large difference in their convex hulls (2.83 and 5.71 respectively). These results suggest that although the two groups were similarly dense, one (the SW cluster) occupied a slightly larger area than the other.

*Int Experiment*

The number of local maxima detected was 26, the optimal number of clusters for these maxima was found to be 1. After clustering, 19 peaks (73.08%) were found to fall in the NW quadrant. The centroid of this cluster was also found to be closest to the NW platform location.

Our shuffled data reveal that on average we would expect this number of peaks to form a single cluster 94% of the time, meaning that our observation does not deviate from what would be expected by chance (p = .94).

However, the single cluster in our real data is significantly smaller and more compact than would be expected by chance, as shown by our point to center and point to point measures respectively [t(2624) = -4.14, p < .001, t(63568) = -17.16, p < .001]. This is because shuffled peaks tend to cover the watermaze surface randomly, whereas our observed peaks are confined mainly to a single quadrant. In support of this interpretation, we also found that our cluster’s convex hull or area (24.52 pixels) was much smaller than the average found in our shuffled data (mean = 59.37 pixels, SEM = 1.71).

**q**PCR

During retrieval in addition to the gene effect shown in the main text, we also found an experiment effect (F=3.9, df 1.3/73, p<0.05). Animals that had experienced a similar trial as the retrieval event already at 24h (Exp A) showed less gene expression changes in *Arc* and *Zif* than animals, who experienced such a retrieval event for the first time at 7d (Exp B).

*Retrieval Induced qPCR*

Various studies using immunocytochemistry have examined IEG expression at the time of memory retrieval after varying ‘consolidation periods’ after training ([Maviel et al. 2004](#_ENREF_31); [Wheeler et al. 2013](#_ENREF_70)). We did not anticipate major differences in the impact of our two main behavioural conditions on retrieval-associated IEG expression at a long time-period after they occurred, but felt it would be valuable to examine whether there were any detectable differences. Accordingly, as a final step in the study, retrieval-associated IEG expression (n=80, including 16 untrained home-cage animals) was measured after a probe test conducted at the same 7-day interval after training (Fig S15). In this case the ‘trace-competition’ design could again not be used and we directly compared retrieval in Sleep and N+SD animals, controlling for extraneous variables (swimming, body temperature etc.). The data are plotted such that positive values indicate higher gene-expression in Sleep and negative values in N+SD. In keeping with our expectation, relatively small differences between the conditions were seen at this 7-day time-point. Expression of *Zif-268* and *Arc* in mPFC was observed to be relatively higher when Sleep closely followed training, with the opposite trend in HPC – but in both cases the differences were less than 20% (Fig. S15). Interestingly, the opposite pattern was seen for *cFos*, with the overall ANOVA showing a Gene x Brain area interaction. This interaction pointed to the possibly greater importance of *cFos* at the time of memory encoding than during retrieval, prompting a direct comparison of encoding and retrieval.

To realise this comparison, we referenced the animals of Fig S15 (retrieval) to a neutral control (see SF16). As seen previously in encoding (Fig. 3) retrieval also showed significantly elevated expression of the three IEGs relative to a control group without watermaze experience, with the highest levels for *Arc* and *Zif-268* (Fig. S16). In absolute terms, expression in HPC was higher than in the mPFC (Fig. S16). Further, we then compared retrieval to the ‘encoding’ animals plotted in Figs 3B,C middle panels. This comparison revealed a significant Gene x Trial type interaction, with subsequent comparisons showing *cFos* elevated relative to the two other genes at encoding, but relatively decreased at retrieval.
